# Progress on Electrolytes Development in Dye-Sensitized Solar Cells

**DOI:** 10.3390/ma12121998

**Published:** 2019-06-21

**Authors:** Haider Iftikhar, Gabriela Gava Sonai, Syed Ghufran Hashmi, Ana Flávia Nogueira, Peter David Lund

**Affiliations:** 1 New Energy Technologies Group, Department of Applied Physics, Aalto University, P.O. Box 15100, FI-00076 Espoo, Finland; haider.iftikhar@aalto.fi (H.I.); peter.lund@aalto.fi (P.D.L.); 2 Laboratory of Nanotechnology and Solar Energy, Chemistry Institute, University of Campinas–UNICAMP, P.O. Box 6154, 13083-970 Campinas, SP, Brazil; gabrielasonai@hotmail.com (G.G.S.); anafla@unicamp.br (A.F.N.); 3 Department of Applied Physics, Aalto Startup Center, A-Grid, Otakaari 5, FI-02150 Espoo, Finland

**Keywords:** dye-sensitized solar cells, electrolytes, hole-transporting materials, charge transfer, printing, copper redox shuttles, cobalt redox shuttles and iodine electrolytes

## Abstract

Dye-sensitized solar cells (DSSCs) have been intensely researched for more than two decades. Electrolyte formulations are one of the bottlenecks to their successful commercialization, since these result in trade-offs between the photovoltaic performance and long-term performance stability. The corrosive nature of the redox shuttles in the electrolytes is an additional limitation for industrial-scale production of DSSCs, especially with low cost metallic electrodes. Numerous electrolyte formulations have been developed and tested in various DSSC configurations to address the aforementioned challenges. Here, we comprehensively review the progress on the development and application of electrolytes for DSSCs. We particularly focus on the improvements that have been made in different types of electrolytes, which result in enhanced photovoltaic performance and long-term device stability of DSSCs. Several recently introduced electrolyte materials are reviewed, and the role of electrolytes in different DSSC device designs is critically assessed. To sum up, we provide an overview of recent trends in research on electrolytes for DSSCs and highlight the advantages and limitations of recently reported novel electrolyte compositions for producing low-cost and industrially scalable solar cell technology.

## 1. Introduction

Climate change mitigation will require a massive switch to clean energy technologies [1,2]. Solar energy is the fastest growing technology among renewable energy sources [1,3,4], with the potential to supply a significant share of the global energy demand in the future [1,3,4]. 

Silicon (Si) solar cell technology currently dominates the photovoltaics (PV) market [5]. This technology has become significantly cheaper during the past 10 years, which has resulted in rapid market-growth. Although the future prospects of crystalline silicon (c-Si) solar cells are promising, issues, such as their complicated fabrication process, long energy payback time, and use of hazardous chemicals, may open up growth-opportunities for other PV technologies in the future. Third-generation PV technologies, such as dye-sensitized solar cells or perovskite solar cells, have high potential for industrial scale manufacturing, as they can be produced while using established scalable manufacturing methods, such as screen printing, inkjet printing, or slot die coating [6,7,8,9,10]. 

The classical n-type dye-sensitized solar cells (typically referred as “DSSCs”) among the other existing third-generation PV technologies have broadly been studied since 1991 after the ground-breaking work that Grätzel and O’Reagan published [11]. DSSCs offer numerous inherent advantages, such as low costs production, facile fabrication schemes, use of cheap and abundantly available materials (e.g., TiO_2_ and carbon-based materials), and the possibility of using scalable material deposition methods in their deposition over traditional fluorine-doped tin oxide (FTO)-Glass substrates and options for translating this traditional rigid device structure over flexible substrates [6,7]. DSSCs also exhibit higher performance under low- and indoor-light conditions than the other photovoltaic technologies [12]. Their other advantages include the diversified possibility to design them with a large number of different materials, with flexibility in shape, colors, and even transparency, allowing for application with promising possibilities, such as in photovoltaic windows and in textiles [8,13,14]. 

The energy conversion efficiency of the lab-sized DSSC has decently increased from 7% in 1991 to the current record of 14.3%, which was obtained in 2015 [11,15]. This impressive improvement in solar-to-electrical conversion efficiency has been achieved through device optimizations, use of transition metal redox couple in combination with suitable dyes, and low viscosity solvents, such as acetonitrile [15,16,17]. In the work that was published by Kakiage et al. in 2015, the authors prepared devices by co-sensitizing the photoelectrodes (PEs) with a carboxy-anchor organic dye (LEG4) and alkoxysilyl-anchor dye (ADEKA-1) in combination with a cobalt-based electrolyte, reaching η = 14.3% under 100 mW cm^−2^ illumination [15]. Cao et al. presented a new DSSC configuration with copper-based electrolyte reaching an impressive energy conversion efficiency (η = 32%) under low light intensity (1000 lux) conditions very recently [18]. These impressive recent improvements in the solar-to-electrical conversion efficiencies have again become reasons for a resurgence in efforts to producing DSSC in large scale, such as large modules for terrestrial power generation and even small modules that focus on portable electronics [19,20].

However, the most efficient DSSC normally use a liquid electrolyte, which is still a limiting factor for the large-scale production. Major commercial success has not been realized despite the existence of a few commercial applications [12,21,22,23,24], due to issues with their long-term performance stability and up-scaling challenges [12,25,26,27,28]. One of the major reasons for the modest long-term stability of DSSCs is their liquid electrolyte, which serves as a mediator between the photoelectrode (PE) and counter electrode (CE) and it has problems that are associated with leakage and exposure to ultraviolet radiations [29,30]. 

This review discusses recent progress on the development of DSSC electrolytes of different compositions and describes the state-of-the-art in relation to the performance and long-term stability of devices while incorporating these electrolytes. Several newly introduced electrolyte materials are also discussed. Finally, the role of electrolytes in future DSSC research and commercialization activities is highlighted.

## 2. Device Structure, Operating Principle and Charge Transport Mechanisms in DSSCs

DSSCs are photoelectrochemical devices, which convert light energy into electrical energy by receiving photons from sunlight that excite the electrons of the dye molecule, followed by their injection into the conducting band of the adjacent mesoporous TiO_2_ layer.

**Figure 1:**
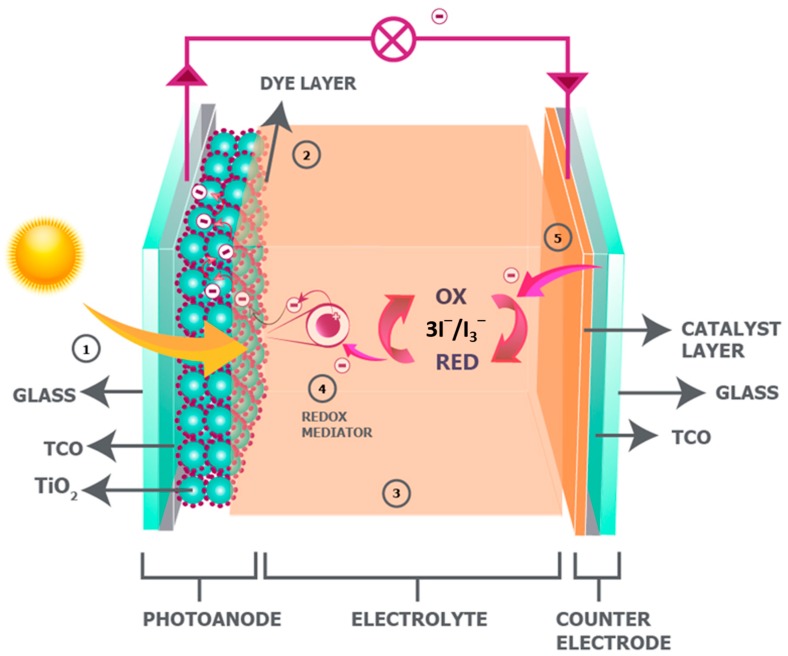
Device structure of a dye-sensitized solar cell employing iodine electrolyte (employing I^−^/I_3_^−^) redox couple) as an example [11,26,29,30].

A traditional DSSC (as shown in Figure 1) typically consists of the following components:

1. A photoanode, which is traditionally fabricated on a transparent conducting oxide (TCO) glass, such as a glass substrate that is coated with indium-doped tin oxide (ITO) or fluorine-doped tin oxide (FTO), by depositing the mesoporous electron transporting TiO_2_ layer on this, either via doctor blading [28,31], screen printing [2,32,33], or inkjet printing [34,35].
2. A monolayer of dye, which is usually based on ruthenium sensitizers, adsorbs over the mesoporous TiO_2_ layer through its anchoring groups [2,11].
3. A liquid electrolyte containing a redox mediator, such as iodide/triiodide along with other additives, including an organic solvent to perform electron exchange during cell operation [2,11].
4. A CE (cathode), comprising a similar TCO-coated conducting glass substrate to that of the photoanode loaded with a catalyst layer, such as Pt or carbon [4,36], which receives electrons from external circuits and reduces the triiodide ion back to an iodide ion through an efficient charge transfer process.

**Figure 2:**
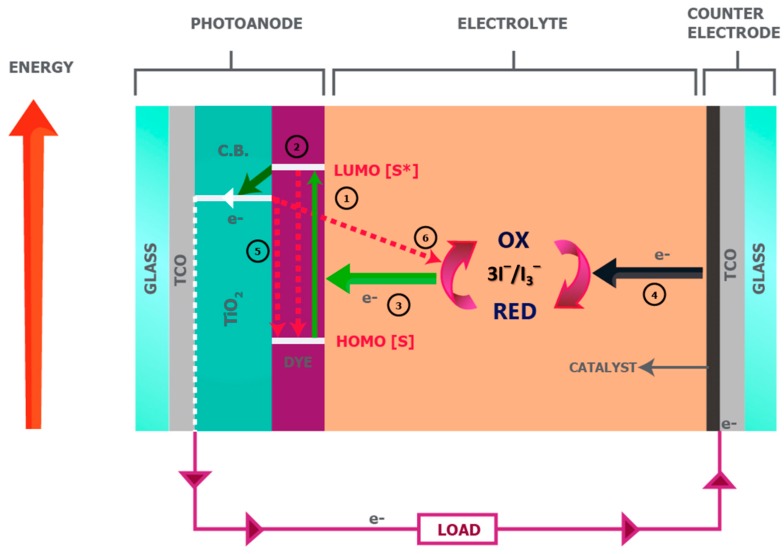
The operating mechanism of a typical dye-sensitized solar cell with iodine electrolyte (employing I^−^/I_3_^−^) redox couple) as an example. 1: Excitation of the dye. 2: Injection of excited electron into the conduction band of the TiO_2_ semiconductor. 3: Regeneration of the dye takes place as a result of electrons accepted from the reduced state of the redox mediator, which in turn becomes oxidized itself in the process. 4: Regeneration of the electrolyte by accepting electrons from the counter electrode and returning to (RE) state [2,38].

Figure 2 illustrates the operational sequence of the DSSC, which begins with the absorption of photons that enter through the transparent photoanode and excites an electron of the dye from a low-energy state (referred to as the highest occupied molecular orbital, HOMO) to a high-energy state, i.e., the lowest unoccupied molecular orbital (LUMO) of the molecule (Equation (1)).

The excited electron from the dye molecule is injected into the mesoporous TiO_2_ layer, leaving the dye molecule in an oxidized state at the TiO_2_/dye interface (Equation (2)). The dye is then regenerated by receiving an electron from the iodide ion of the redox couple of the liquid electrolyte, which oxidizes into a triiodide ion and then propagates towards the CE of the DSSC (Equation (3)). Finally, the triiodide ion is regenerated at the CE by receiving the returning electron from the external load through an efficient catalyst layer, i.e., Pt (Equation (4)), to complete the cycle [4,11,14,36,37,38].

Two other reactions occur, in addition to the abovementioned sequence. These are known as recombination reactions or dark reactions. The first dark reaction corresponds to the recombination of those electrons that were excited to the LUMO and injected into the mesoporous titania layer, back into the oxidized dye (Equation (5)). The second dark reaction corresponds to the recombination of the same injected electrons in the titania layer with the oxidized triiodide ion of the electrolyte, which reduces it back to its original iodide ion form (Equation (6)). Both these dark reactions occur at a much slower rate than the forward reactions, and consequently DSSCs function as relatively efficient PV devices.

A primary focus of research on DSSCs has been on designing optimized individual components to achieve improved performance. Electrolytes are a key component of DSSCs for this, since *V_OC_* is determined by the difference between the Fermi-level of the semiconducting oxide (for example TiO_2_) and the Nernst potential of the used redox species within the electrolyte [2,11,39]. Moreover, the electrolyte and its composition play a vital role in defining the performance of various device designs, since DSSCs can be fabricated with numerous configurations [30]. Several popular architectures of DSSCs are discussed in the following, and the role of the electrolyte in these architectures is described in detail.

Dye excitation

(1) $$S\left[{\mathrm{TiO}}_{2}\right]+\mathrm{hv}\underset{\;}{\to }{\;S}^{*}\;\left[{\mathrm{TiO}}_{2}\right]$$

Electron injection

(2) $$S^{*}\;\left[{\mathrm{TiO}}_{2}\right]\underset{\;}{\to }{\;S}^{+}\left[{\mathrm{TiO}}_{2}\right]+{\;e}^{-}\left[{\mathrm{TiO}}_{2}\right]$$

Dye regeneration/Mediator oxidation

(3) $$S^{+}\left[{\mathrm{TiO}}_{2}\right]+\;\mathrm{Red}\;\underset{\;}{\to }\;S\left[{\mathrm{TiO}}_{2}\right]+\mathrm{Ox}$$

Mediator regeneration

(4) $$\mathrm{Ox}+{\;e}^{-}\left[\mathrm{CE}\right]\underset{\;}{\to }\;\mathrm{Red}\;+\;\mathrm{CE}$$

Dark reaction—Dye recombination

(5) $$e^{-}\left[{\mathrm{TiO}}_{2}\right]+{\;S}^{+}\left[{\mathrm{TiO}}_{2}\right]\;\underset{\;}{\to }\;S\left[{\mathrm{TiO}}_{2}\right]$$

Dark reaction—Recombination due to Ox

(6) $$e^{-}\left[{\mathrm{TiO}}_{2}\right]+\mathrm{Ox}\;\underset{\;}{\to }\;\mathrm{Red}$$

## 3. Configurations of DSSCs and the Role of Electrolytes

**Figure 3:**
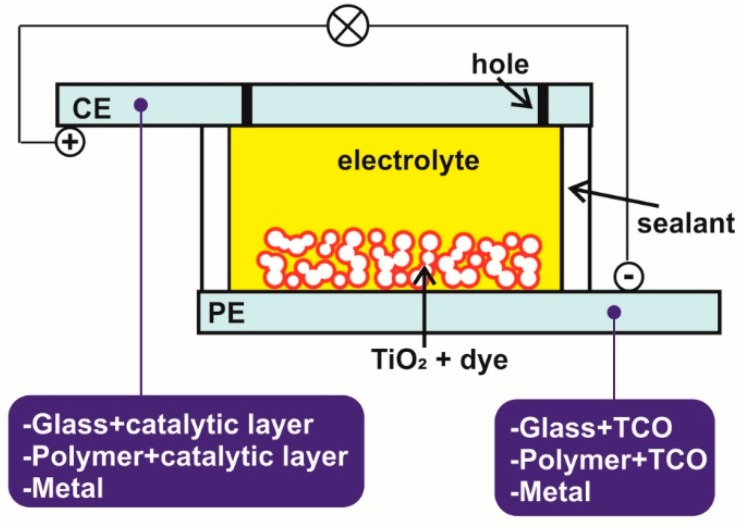
Schematic illustration of the different possible configurations of dye-sensitized solar cells (DSSCs) [29,30].

Traditional DSSCs were first fabricated while using TCO-coated glass-based PEs and TCO-coated glass-based CEs by integrating a solvent-based iodide-triiodide redox couple electrolyte (Figure 1) [11]. The best device efficiencies for DSSCs have all been reported with this device design (Figure 3) with a low viscosity solvent, such as acetonitrile (ACN). Advantages over other types of DSSCs [4,29,36,40] include little to no light attenuation and no direct absorption of light in the electrolyte layer before reaching the dye molecule that was anchored to the TiO_2_ nanoparticles. The highest certified efficiency reported to date with this device structure is 11.18% [41] for an iodide/triiodide mediator in conjunction with an organic sensitizer LEG4 and 14.3% [15] for a cobalt-based mediator employing ADEKA + LEG4 co-sensitizing dyes, both under full sunlight illumination. 

Nonetheless, both the solvent and the iodide-triiodide redox couples have limitations together, such as limited *V_OC_* due to their redox potential, and corrosive behavior when integrated with metal-based substrates in DSSCs [42,43,44,45,46,47,48].

Low boiling point solvents, i.e., ACN (acetonitrile CH_3_CN) or valeronitrile, have other problems, which include the leakage of electrolytes from the DSSC device structure, which has been observed in harsh long-term stability tests [25,26,27,28,29], and their incompatibility with conducting polymer substrates (such as ITO-PET (polyethylene terephthalate) and ITO-PEN (polyethylene naphthalate)) that are used in flexible DSSCs [29,30,49,50,51].

Hence, the performance of DSSCs under different operating conditions is highly dependent, not only on device structure, but also on the selected electrolytes and their corresponding configurations. Some popular DSSC configurations are briefly discussed in the following sections. 

### 3.1. Bifacial Semi-Transparent and Front-Illuminated DSSCs on Rigid and Flexible Substrates

#### 3.1.1. Bifacial and Front-Illuminated DSSCs on Rigid Substrates 

Traditional DSSCs (as discussed in previous sections) that were fabricated on transparent or rigid FTO-glass substrates can be classified as either bifacial or front-illuminated. In one of the simplest examples, transparent FTO-glass coated with a semi-transparent and dye-sensitized TiO_2_ layer serve as front-illuminated and transparent PE (Figure 3). 

On the other hand, transparent FTO-glass that was loaded with a highly transparent Pt catalyst layer, which functions as a CE, can also be used as a reverse-illuminated window [14,52]. Front illumination, i.e., the illumination from the PE side, nevertheless, has an inherent performance advantage over reverse illumination from a CE due to the almost negligible absorption of sunlight in the FTO layer before hitting the dye-coated TiO_2_ layer. In contrast, in reverse illumination, sunlight is typically absorbed by some of the active components of the DSSC before exciting the dye molecule of the PE, including fractional absorption in the FTO layer and in the Pt or alternative semi-transparent catalyst layer, and significant absorption in the electrolyte layer. In this regard, light management and the transparency of the active layers are the vital determinants of the performance of reverse-illuminated DSSCs. Despite this limitation, the traditional glass-based bifacial configuration has been keenly investigated due to the potential for integrating such aesthetic PV applications into modern buildings [52]. There have been some recent commercial demonstrations of artistic colourful DSSCs for building-integrated photovoltaics (BIPV). However, studies are needed on suitable electrolytes for these, and regarding the long-term stability and performance of such installations [53,54,55].

One additional drawback of rigid bifacial DSSCs is the fact that their device efficiencies remain lower than those of the conventional front-illuminated DSSCs, due to the absence of an opaque scattering TiO_2_ layer [33,56]. Such a layer cannot be used in transparent device architectures for building applications, and it may only have limited use for rooftops and consumer electronics applications. The highest device efficiencies that have achieved to date have been reported for front-illuminated DSSCs with a light-scattering TiO_2_ layer [15,16,17,19]. However, iterestingly, both bifacial and front-illuminated DSSC device designs are among the most stable device structures when tested with alternative solvent-based electrolytes as compared to traditional electrolytes based on low viscosity solvents (i.e., ACN or valeronitrile) [25,26,27,57,58].

#### 3.1.2. Bifacial and Front-Illuminated DSSCs on Polymer Substrates 

In addition to the factors that are discussed above for rigid DSSC configurations, the overall performance of flexible DSSCs, whether front- or reverse-illuminated, also depends on the opaqueness and transparency of an electron selective layer that was based on low temperature-deposited TiO_2_ nanoparticles [59,60]. Flexible plastic DSSCs require low temperature (<200 °C) processing of the associated materials for both the PEs and CEs due to their deformation above 150–200 °C [29,30]. Hence, a high temperature sintering step at (500 °C) cannot be used to produce semi-transparent TiO_2_, as is used for glass substrates [59]. Hence, producing stable and highly transparent TiO_2_ on transparent and flexible conducting polymers (such as ITO-PET or ITO-PEN substrates) is a major challenge in this research field.

However, a transparent flexible PE can be achieved by depositing a layer of a low temperature and binder free TiO_2_ nanoparticle paste on highly transparent ITO-PEN substrates, followed by either mechanical or cold isostatic pressing [59,60]. Pressing promotes interparticle connectivity and it may also enhance the transparency of the fabricated PE on these conducting polymer substrates [59,60]. On the other hand, semi-transparent flexible CEs have also been demonstrated via the low temperature deposition of Pt nanoparticles based layer by sputtering, by chemical, or electrochemical deposition schemes [61,62,63].

Other issues of flexible DSSCs include the chemical stability of electrodes in the presence of harsh solvents, such as ACN, as well as the negative effects of ultraviolet radiation on the transparency of the front electrode (PE), resulting in performance deterioration over time. 

The PE of a plastic DSSC usually comprises an ITO layer as the TCO on a polymer substrate (such as PET or PEN), which is typically coated with a low temperature TiO_2_ layer [59,60,64,65]. This low temperature processed TiO_2_ layer is also sensitized with a ruthenium sensitizer. The persistent challenge of flexible PEs on polymer sheets is to produce a low temperature processed titania layer of similar performance to that achieved with high temperature processed titania layers on glass.

The high temperature doctor bladed or printable paste of TiO_2_ that is used for glass or metal substrates typically consists of titania nanoparticles mixed with an organic binder, such as ethyl cellulose, and terpineol, a viscous solvent, which are removed via the sintering of the electrodes at 450–500 °C [33,66]. This high temperature sintering of the deposited titania layer on glass or metal electrodes not only removes the organic binders and viscous solvents, but also strongly promotes the interparticle necking effect, which enhances the electron conduction of the PE. 

However, due to temperature limitations, these organic binders and high boiling point viscous solvents cannot be used for the deposition of titania paste over the conducting polymer substrates [67]. This severely affects the quality and performance of low temperature processed titania layers, which also exhibit poor mechanical stability on flexible substrates [67].

The traditional semi-transparent catalyst layer of Pt nanoparticles for CEs can be deposited via numerous low temperature routes such as chemical platinization [68,69] electrochemical platinization [40] sputtering [70], screen printing using low temperature Pt pastes for roll 2 roll processing [71], and inkjet printing [30] in contrast to the limited choices for producing an efficient PE on polymer substrates.

Furthermore, alternative catalyst materials than Pt can also be chosen, such as low temperature carbon composites [72,73,74], carbon nanotubes [49,75,76,77], graphene flakes [78,79,80,81], or conducting polymers, such as PEDOT:TsO and PEDOT:PSS [75,82,83].

Interestingly, merging dry-printed single-walled carbon nanotubes (SWCNT) [84] with transparent alternative redox couples, such as cobalt shuttles, appears to be a promising new approach to producing bifacial polymer DSSCs. These SWCNTs have already been tested as metal-free CEs on polymer substrates with a mechanical transfer process and they have been assembled in a lab-sized DSSC configuration with glass PEs [85]. These transparent metal-free CEs may help to reduce both photonic and resistive losses that occur in reverse illumination of flexible bifacial DSSCs, since the transparencies of SWCNTs can be tuned without significantly compromising the sheet resistances. Figure 3 represents a schematic of a polymer bifacial DSSC.

### 3.2. Reverse-Illuminated DSSCs

Sunlight enters and excites the dye molecule via the CE, which is also known as a window electrode, when the PE of a DSSC is fabricated over an opaque metal substrate, instead of a glass or polymer substrate. This device architecture is often referred to as a reverse-illuminated DSSC [29,40,42,43,69].

Since a high temperature sintering step can be applied to metals, this configuration has the key advantage of high-performance PEs, which can be assembled with transparency-optimized CEs to achieve excellent PV performance. Another advantage of this configuration is that it has less resistive losses in the PE, when compared to many other substrates, since the metals are more conductive than ITO-glass, FTO-glass, ITO-PET, and ITO-PEN sheets. 

The disadvantages of this configuration include the reverse illumination itself, by which the light gets absorbed by the semi-transparent catalyst layer as well as by the electrolyte layer in between the PE and CE of the cell. 

Their corrosion in the presence of the iodine and cobalt based redox shuttles is another limitation for these metal substrates, which severely declines the initial performance. Titanium has remained the number one choice in fabricating metal based reverse illuminated DSSCs due to this limitation, since it exhibits excellent resistance against corrosion [40,86,87,88,89]. The other metals substrate reported to produce reverse illuminated DSSC is Inconel. However, reports are few, and this substrate requires further investigation. Most other metals tested, such as copper and stainless steel (StS), are susceptible to corrosion and have been unable to maintain stable PV performance in long-term stability tests [29,42,68].

A further challenge for the production of efficient PEs whike using some of these alternative metal substrates, such as StS, which are inexpensive when compared to titanium metal sheets, is the high temperature sintering step, which affects the inherent properties of alloys of these metal substrates. Inexpensive metal substrates may be oxidized upon heating to extreme temperatures, which reduces the PV performance of the fabricated devices [42,43].

Again, electrolyte selection plays a key role in designing stable and efficient reverse-illuminated metal-based DSSCs. Reverse-illuminated DSSCs with a metal PE can br currently realized either in combination with a glass CE or a flexible polymer CE. 

Figure 3 also represents the schematic of a DSSC based on a metal PE and glass CE [29,30,42,43], which offers some advantages, such as high temperature sintering of the glass-based CEs. Hence, a traditional glass-based CE loaded with thermally reduced Pt nanoparticles can be assembled with a metal-based PE. High transparency glass CEs can be achieved not only with a Pt nanoparticle-based catalyst layer, but also with high-temperature-processed graphene nano-flakes [78,79,80], which are an alternative choice for the optimization of reverse-illuminated DSSCs. However, roll-to-roll production cannot be realized if rigid glass-based CEs or a rigid metal-based Pes are used (at least if the thickness of the metal substrates used remains the same that in traditional glass-based electrodes, i.e., 2–4 mm).

Adopting flexible metal-based PEs and flexible transparent CEs enables fully flexible reverse-illuminated DSSCs to be rapidly produced with roll-to-roll fabrication schemes. This flexible and reverse-illuminated configuration is a viable choice for upscaling to an industrial level since these DSSCs also work efficiently at low light intensities. Commercial players, such as G24 Power [22], have produced several consumer electronic products that which are powered by this flexible and reverse-illuminated DSSC configuration constructed with a titanium-metal-electrode-based PE and a conducting polymer (ITO-PET) CE. The highest efficiency that was reported for a reverse-illuminated DSSC so far (8.6%) was for a device fabricated with a PE based on flexible stainless steel (StS) and plastic CEs [68]. However, no stability data for these devices was reported. The same configuration can also be produced by integrating all the possible transparent flexible CEs, e.g., ITO-PEN and ITO-PET, substrates in combination with other metal and opaque polymer PEs. Additionally, Figure 3 represents the schematic illustration of fully flexible reverse-illuminated DSSCs.

### 3.3. Metal Counter Electrode-Based DSSCs

Due to the temperature limitations for inexpensive metal substrates that were discussed in the previous section, DSSCs with metal-based CEs utilize low-temperature methods for the preparation of the catalyst layer, such as sputtering of Pt [46,88] or low temperature-based chemical platinization [68,69,71,90]. These low temperature-based catalyst layers have given comparable performance to that achieved with the high temperature processed Pt catalyst layers. High-temperature methods over metal substrates are believed to increase the surface oxidation of the metal surfaces, thereby decreasing the overall charge transfer [29,48].

Metal CEs may also provide better stabilities when compared to ITO-PET or ITO-PEN, where the ITO layers may partially or completely dissolve upon exposure to severe chemical treatments in cases where acids or other harsh solvents are employed during fabrication [91].

Hence, the selection of electrolytes again plays a vital role in achieving optimal performance from metal CE-based DSSCs. Another key factor is the optimization of the catalyst layer. This should not only be compatible with the metal electrode, but also provide a high resistance to metal corrosion in the presence of corrosive electrolytes. Metal-based CEs are subject to degradation with conventional tri-iodide redox electrolytes, owing to their function as cathodes [29,44].

Similarly, to reverse-illuminated DSSCs, metal CE-based DSSCs can also be categorized into those that are based on a rigid metal CE and a rigid glass PE [29,30,42,43] or a flexible metal CEs and a flexible polymer PE [29,30,45].

DSSCs that were based on a rigid metal CE and rigid glass PE (Figure 3) are similar in configuration to traditional DSSCs based on a glass PE and glass CEs, but they can only be illuminated from the front via the transparent glass PE. They cannot be illuminated from the back due to the opaqueness of the thick (2–4 mm) metal CEs. In the fully flexible DSSCs, the flexible PE is typically fabricated over a flexible conducting polymer by employing low temperature TiO_2_ paste and this is combined with a flexible and opaque thin metal CE. The working principle of these flexible DSSCs is similar to that of rigid metal CE-based DSSCs.

Special care is needed to choose a suitable electrolyte that is minimally degrading the metal-based CE for these non-conventional (not glass) CEs and PEs and is also compatible with the polymer PE, without causing any leakages or water uptake. Such DSSC configurations have rarely been reported [46,68,71,90], possibly due to the superior performance of low temperature titania-based flexible PEs on conducting polymer substrates, i.e., ITO-PETs and ITO-PENs.

### 3.4. Monolithic DSSCs

**Figure 4:**
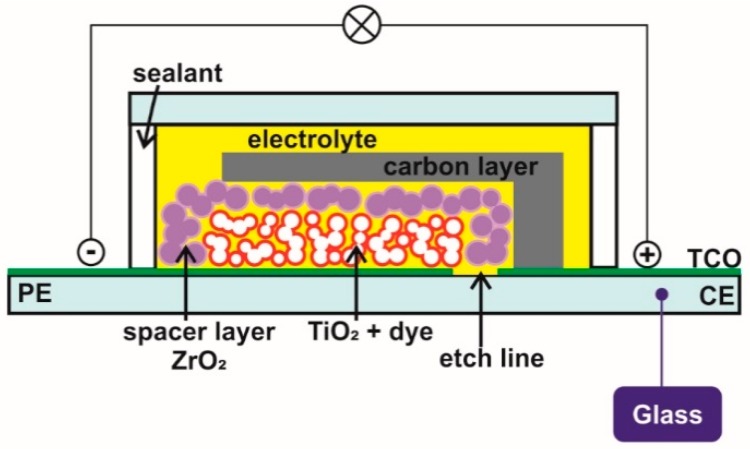
Illustration of a typical assembly of a monolithic DSSC with all the key components [25,92,93,94,95].

The monolithic DSSC cell design is, by far, the most different to the conventional sandwich configuration, both in terms of its materials and assembly (Figure 4) [25,92,93,94]. The entire cell is built on a single substrate, which is its most striking feature, as the CE is on the top of the PE [25,92,93,94]. The PE is fabricated by the usual TiO_2_ deposition, followed by the printing of an insulating porous spacer layer (typically composed of ZrO_2_) over it to avoid physical contact between the PE and CE [25,92,93,94]. 

The catalyst layer of the CE generally comprises a thick porous carbon layer. Porosity not only facilitates charge transfer, due to a high surface area, but it also enables the application of an electrolyte without drilling holes [25,92,93,94]. The carbon film must have a good enough sheet resistance, as it is the sole conducting layer on the CE and it serves as a low cost and effective alternative to traditional Pt layers. The monolithic cells generally exhibit lower efficiencies than traditional DSSC configurations, since their insulating spacer hinders the diffusion of ions [25,92,93,94]. 

Careful choice of the electrolyte in this configuration is again vital. Traditional solvent-based electrolytes may damage the polymer sealants that hold the cover glass substrate. Moreover, extremely volatile solvents, such as ACN-based electrolytes, cannot be used due to their possible leakage at extreme temperatures (80–90 °C). Hence, durability requirements dictate the need for electrolytes without solvents and volatile components, as previously discussed for polymer bifacial DSSCs.

### 3.5. Solid State DSSCs

The solid state DSSCs configuration is actually a modified form of conventional DSSCs, where a liquid electrolyte has been replaced with a solid state hole-transporting material (HTM) or solid polymer electrolyte to avoid the leakage problem [30,31,96,97,98]. The HTM, which is an organic semiconductor material, is typically fabricated from its precursor solution on the top of the sensitized mesoporous TiO_2_ film [97,98]. This solution-processed HTM deposition appears to be highly compatible with roll to roll (R2R) processing, especially for the fabrication of flexible solid state DSSCs [99].

Using an HTM could also provide better diffusion characteristics for solid state DSSCs than conventional DSSCs, since extremely thin layers (10–50 nm) are sufficient. Conventional DSSCs exhibit high diffusion resistance when viscous ionic liquid electrolytes are used, and they are dependent upon the optimization of the thickness of the Surlyn or Bynel separator frame foil. The solid-state configuration has an inherent edge when compared to traditional DSSCs, since it is not dependent on separator foil, and hence has lower diffusion resistance [31,96,97,98]. Nevertheless, the long-term device stability of this promising configuration looks challenging to that of conventional configurations containing liquid electrolytes and it has been rarely reported with few HTMs [100,101,102,103].

Nonetheless, the production cost of solid state DSSCs may be high, due to the high cost of some traditional HTMs, such as 2,2′,7,7′-tetrakis(N,N-di-p-methoxyphenylamine)-9,9′-spirobifluorene (spiro-MeOTAD), and also due to the gold contact, which is typically fabricated over the HTM for efficient collection of the holes that were generated in the device. 

**Figure 5:**
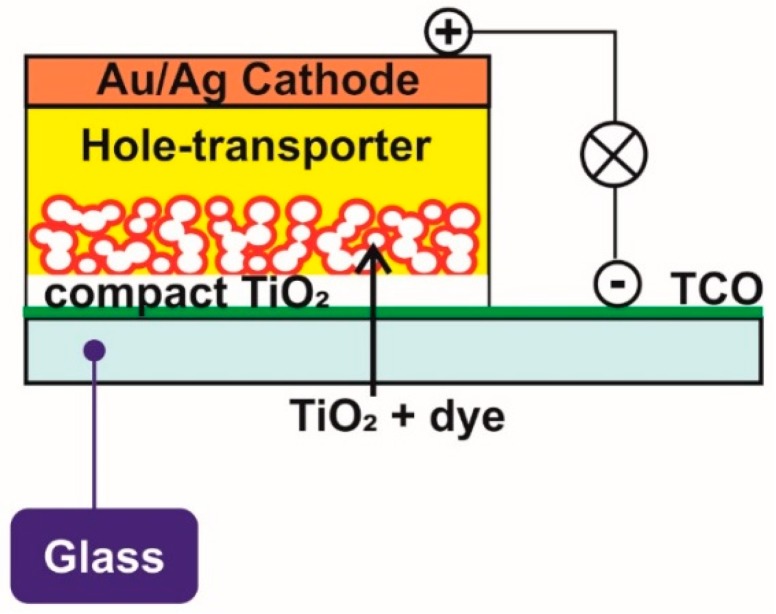
Illustration of a typical assembly of a solid state DSSC cell with a hole transporting material [97].

Figure 5 represents the schematic of a solid state DSSC. Table 1 summarizes the best efficiencies achieved so far for each type of DSSC configuration employing specific electrolytes.

## 4. Electrolytes for DSSCs

As discussed in earlier sections, the electrolytes in DSSCs are responsible, not only for the regeneration of the dye impregnated on mesoporous TiO_2_, but also for charge transport between the PE and CE of the solar cell. 

**Figure 6:**
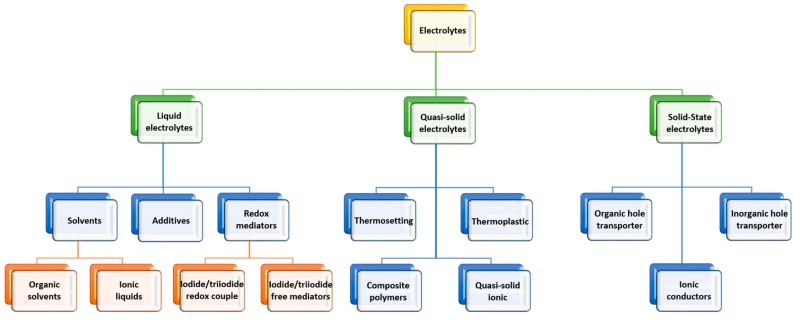
Classifications of electrolytes developed for DSSCs.

Numerous efforts have been made to design novel and efficient electrolyte formulations in order to achieve optimal performance in third-generation solar cells. These electrolytes can be categorized as liquid electrolytes, quasi-solid electrolytes, and solid-state conductors. Figure 6 represents a classification of all the electrolytes that have been developed for DSSCs and they are discussed in detail in the following sections.

### 4.1. Liquid Electrolytes

The first ever DSSC with 7–8% efficiency was reported with a liquid electrolyte that consisted of an organic solvent containing an iodide/triiodide redox couple [11]. No additional additives were used in this electrolyte solution. Later, many electrolyte formulations with additional additives and unique solvents have been produced. Some of these have achieved high DSSC performance along with improved long-term PV performance stabilities [26].

The use of a liquid solvent-based electrolyte offers numerous advantages over other types of electrolytes, such as low viscosity, high conductivity, excellent connectivity interaction at the electrode/electrolyte interface, and, most importantly, simple preparation methods, thereby rendering high conversion efficiency [16,109]. Interestingly, DSSCs with liquid electrolytes and a traditional device configuration demonstrate the highest conversion efficiencies so far, approaching >14% under full sunlight illumination [15]. The main limitation for low viscosity liquid electrolytes is leakage from the cell assembly, which decreases the initial PV performance. Several moderate viscosity solvents have been introduced to compensate for this, which have demonstrated impressive long-term performance stability [26,57,58,110,111,112].

As illustrated in Figure 6, the liquid electrolytes for DSSCs include those based on organic solvents, ionic liquids, iodide-/triiodide-free mediators, and iodide/triiodide redox shuttles. These sub-classes of liquid electrolyte are discussed in detail in the following sections.

#### 4.1.1. Organic Solvent-Based Electrolytes

Organic solvents provide a phase for the dissolution and diffusion of ionic species, enabling ion transport through the electrolyte. Such solvents in DSSCs should have low cost, low toxicity, and low light absorption [113,114]. They should also be inert towards the dye and be of poor solubility to the sealant materials [112]. Their acceptable melting point should range from −20 °C to 100 °C for their survival under extreme conditions outdoors [112]. They should also exhibit ample chemical stability under both the dark and irradiance conditions, thereby providing a wide electrochemical window, to diminish the degradation of both the cathode and anode operating at their desired electric potential ranges [14,37,112,115]. A good solvent should also have a high enough dielectric constant to provide sufficient dissolution of the ionic salts and low viscosity, so that the diffusion coefficients of the redox mediators are high enough to provide good ionic conductivity in the electrolyte [2,14,36,37]. Mixtures of solvents, selected for their physical and chemical properties, are used to achieve optimal performance of DSSCs, since no single solvent has all of these properties [57,58,111]. 

The intrusion of water and moisture are key factors that cause performance degradation in DSSCs [112,113,114]. However, interestingly, adding 20% water to a non-aqueous electrolyte has been shown to result in a 0.2% increase in conversion efficiency without losses in long-term stability [113]. Water and various alcohols are lower in chemical stability for long-term application and are therefore unsuitable as prime solvents. However, in recent studies, water has emerged as a promising solvent to prepare aqueous-based electrolytes for DSSC [116,117,118,119]. Water being used as the main solvent presents some advantages, such as producing a low cost, nontoxic, non-flammable, and an eco-friendly photovoltaic devices [116,117,118,119,120]. The efficiency record for a 100% water-based DSSC is 5.97%, as obtained by Lin et al. in 2015, in combination with a metal-free organic dye (EO3) and TEMPO/iodide electrolyte [121]. Most of the studies in this research area present energy conversion efficiency usually below 3% device despite this impressive efficiency presented by Lin for an aqueous-based DSSC [116,117,118,119,120]. Accordingly, further improvements on this aqueous system, as well for the other components of DSSC, are necessary.

Among the organic solvents, ACN remains the foremost choice of a liquid electrolyte, owing to its outstanding solubility, exceptionally low viscosity, and remarkable chemical stability. Its electrochemical window of >4 V [115] has resulted in the best device efficiency (>14%) for DSSCs to date [15]. However, it has inherent stability issues due to its low boiling point (82 °C) and rapid evaporation when exposed to stressful conditions [112]. However, the toxicity of ACN means that it is not very suitable for consumer electronics applications. Nonetheless, tt does offers possibilities to produce proofs of concept for testing new dyes and catalyst-based DSSCs systems due to its ability to accomplish the photochemical processes without mass-transport limitations [122]. 

Although ACN has proven to be the prime choice of fabricating high efficiency DSSCs under full sun conditions [15,16,17,19], evidence of the long-term and high temperature stability of ACN solvent-based electrolytes for high efficiency DSSCs has rarely been reported, as in Table 2.

In addition to ACN, other nitrile solvents with higher boiling points and lower toxicities, such as methoxyacetontrile (MAN) and 3-methoxypropionitrile (MPN), have been widely investigated and used as alternatives to traditional electrolytes [26,36]. 

MPN-based electrolytes have shown impressive long-term PV performance stabilities in DSSCs when subjected to full sunlight soaking at 55–60 °C due to their moderate boiling point and good chemical stability [26].

Other solvents with higher boiling and melting points, such as ethylene carbonate (EC) [11,123], propylene carbonate (PC) [124,125], γ-butyrolactone (GBL) [57,124], and N-methyl-2-pyrrolidone (NMP) [124,126], have also been used to formulate stable electrolytes for DSSCs. 

Some compounds, such as EC or N-methyloxazolidinone (NMO), have melting points that are high enough (15–36 °C) to be within the operational range of DSSCs [36]. For these, the addition of other solvents is required to produce a mixture with a lower freezing point [36,123,127]. The first ever DSSC device actually employed a mixture of EC−AN (80%:20% vol) solvents [11,36].

If necessary, the opposite can also be done, i.e., the boiling point of the final solvent can be increased and thereby optimized by adding a high boiling point solvent to a lower boiling point solvent. Such formulations have demonstrated very high efficiency (>10% under full sun illumination), along with good long-term stability when integrated in the traditional DSSCs [127].

GBL solvent-based electrolytes, whose advantageous characteristics include a low melting point (−44 °C), very high boiling point (204 °C), and favourable viscosity (1.7 cP), have been frequently used in long-term DSSC stability tests [57]. For example, DSSC modules incorporating GBL solvent within the electrolyte were observed to operate for up to 2.5 years under outdoor conditions in one of the longest stability tests reported [57]. Table 2 summarizes the best efficiencies that were reported for various DSSCs employing organic solvent-based electrolytes. 

#### 4.1.2. Ionic Liquid-Based Electrolytes

Electrolytes based on non-volatile and solvent-free ionic liquids (ILs) have been widely investigated for DSSCs. Their promising properties include chemical and thermal stability, moderate ionic conductivity, and minimal vapor pressure [131,132,133,134,135,136,137,138,139,140,141,142]. ILs can be categorized as room temperature ionic liquids (RTILs) [131,133,134,135,141] maintaining low viscosity and a lower melting point (<100 °C) [136,137,138,139,140] and ILs with high melting points and high viscosities. The elimination of the risk of leakage from the cell channel is the main advantage of IL-based electrolyte, which destroys the long-term operational stability of DSSCs. In fact, the first ever stable DSSC was demonstrated with an IL-based electrolyte (containing methyl-hexyl-imidazolium iodide, MHImI), which showed no performance degradation [140].

Ionic liquids possess both anions and cations. The cations can be either ammonium/phosphonium salts or heteroaromatics with low symmetry, weak intermolecular interactions, and low charge densities [36]. The anions can be either halides, pseudohalides anions, or some complex anions, such as borates or triflate derivatives [36]. 

After the first report of a stably operating DSSC, imidazolium salts and various other ionic liquids were used extensively as alternative electrolyte solvents for DSSCs to further improve stability [143]. Much later, a solvent-free ionic liquid electrolyte-based SeCN^−^/(SeCN)^3−^ redox couple was reported, with low viscosity and higher conductivity. 1-ethyl-3-methylimidazolium selenocyanate (EMImSeCN) attained an exceptional energy conversion efficiency of 7.5−8.3% under full sunlight illumination [144]. Nevertheless, devices that were fabricated with this electrolyte did not exhibit good long-term stability. 

In an alternative approach, a ternary melt ionic liquid was used in combination with an alternative ruthenium sensitizer (Z907Na). This combination not only lowered the melting point of the final electrolyte, but it also provided a much-needed reduction in the mass-transport limitation. The performance of the resulting DSSC significantly improved, giving rise to an impressive conversion efficiency of over 8% under AM 1.5G full sun illumination and much improved long-term stability [141].

Many other ionic liquids, including ammonium [145], guanidinium [146,147], phosphonium [148,149], pyridinium, and sulfonium [150,151,152], have also been investigated for their potential use as solvent-free electrolytes in dye-sensitized solar cells. Nonetheless, these have not yielded good efficiencies, owing to their high viscosity as well as mass-transport problems [148,149,150,151,152].

Further improvements in the solar to electrical conversion efficiency of DSSCs was achieved by incorporating a low-viscosity tetrahydrothiophenium melt ionic liquid in the electrolyte [153]. The successful use of non-imidazolium ionic liquids has been demonstrated by mixing the binary melts of S-ethyltetrahydrothiopheniumiodide along with S-ethyltetrahydrothiophenium tricyanomethide or dicyanamide in DSSCs, achieving relatively high power conversion efficiencies (PCEs) of 6.9% and 7.2%, respectively [153]. 

The diffusion coefficient of the mediators in pure ionic liquids is 10-100 times lower than in organic solvents. Their relatively high viscosity and low ion mobility limit the transportation of the mediators for the restoration of the oxidized dye, especially at high illumination intensities [36,154]. Triiodide diffusion becomes a limiting factor in highly fluidic imidazolium dicyanamide ionic solvents at lower temperatures, whereas at higher temperatures, the recombination reactions limit the performance of the ionic liquids [155]. 

A mix of ILs with organic solvents has frequently been used to address the aforementioned problems, which suppresses mass transport limitations without compromising the long-term stability of DSSCs [26,57,58].

Interestingly, optimized concentrations of ILs, such as imidazolium iodides, can also contribute to the effective reduction of the dye molecule, which increases DSSC performance [156]. By combining organic solvents with optimized concentrations of ILs, very impressive (>10%) efficiency under full sun illumination has been successfully demonstrated [128,129], Table 2. 

The mass transport limitation that was exhibited by pure ionic liquids has been resolved by combining imidazolium iodides with high fluidity ionic solvents [157]. A study utilizing such a mixture of a low viscosity ionic liquid electrolyte EMImSCN mixed with PMImI produced an impressive PCE of 7% with a triiodide diffusion coefficient that was 1.6 times higher than that of a pure PMImI electrolyte [158]. 

Table 3 presents the best device efficiencies that were achieved using different IL-based electrolytes and the long-term device stabilities of the fabricated DSSCs.

#### 4.1.3. Alternative Redox Mediator-Based Electrolytes

As discussed in earlier sections, the redox couple is the fundamental component in the electrolytes within DSSCs, being responsible for both dye regeneration and ionic transport between the PE and CE.

Therefore, selecting an efficient, non-corrosive, and suitable redox (electron-transfer) mediator is perhaps the most important step towards the commercialization of high efficiency DSSCs [114]. Currently, the conventional iodide/triiodide redox shuttle remains the most common choice for efficient DSSC electrolytes. These have produced PCEs of up to 11.9% (certified) under full sun illumination [164,165]. 

However, the prime limitation of the iodide/triiodide redox shuttle is its inherent lower redox potential, which prevents a high open-circuit voltage (*V_OC_*) from being achieved [16]. The energetic mismatch between the redox couple and the (S^+^/S) state of sensitizers leads to large potential loss [16]. 

The reaction of iodide to tri-iodide conversion during the dye regeneration is complicated due to the transfer of two electrons during the reaction when using iodide/triiodide redox mediator. The mechanism for the properly reduction of the oxidized dye by the iodide based redox mediator is presented in Equations (7)–(11) [14,166,167].

(7) $$S^{*}\;\underset{\;}{\to }S^{+}+{\;e}^{-}\left[{\mathrm{TiO}}_{2}\right]$$

(8) $$S^{+}\;+{\;I}^{-}\underset{\;}{\to }\;\left(S\cdots I\right)$$

(9) $$\;\left(S\cdots I\right)\;+{\;I}^{-}\underset{\;}{\to }\;\left(S\cdots I_{2}^{-•}\right)$$

(10) $$\left(S\cdots I_{2}^{-•}\right)\;\underset{\;}{\to }S\;+I_{2}^{-•}$$

(11) $$2I_{2}^{-•}\;\underset{\;}{\to }I_{3}^{-}+I_{\;}^{-}$$

Initially, the electron of the excited dye is injected into the conduction band of the TiO_2_ semiconductor (Equation (7)). The electron transfer from the iodide to oxidized dye occurs as a one-electron transfer reaction, leading to the formation of the intermediate complex (Equation (8)). The next step is the addition of another iodide to form the intermediate complex with I_2_^−•^ radical (Equation (9)), which is more thermodynamically favourable instead of a reaction reaching to an iodine atom. The intermediate is dissociated into reduced dye and the diiodide radical, I_2_^−•^ (Equation (10)). Subsequently, the formed I_2_^−•^ is followed by its conversion into triiodide and iodide (Equation (11)) [1,2,3]. The iodide/triiodide redox mediator presents a large driving force for the dye regeneration as a result of these complex regeneration kinetics, and consequently limiting the photovoltage of devices [168].

Another disadvantage that si associated with an iodide/triiodide redox shuttle is its corrosive nature towards metals, such as Ag, Cu, Al, and StS, which restricts its deployment in metal-based DSSCs [30,42,43,47,48].

**Figure 7:**
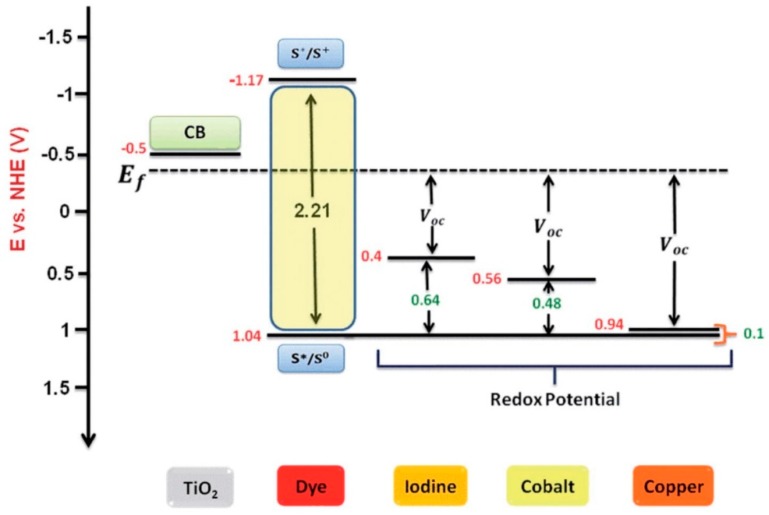
Illustration of energy level of photoanode and different redox mediators: I^−^/I_3_^−^, [Co(bpy)_3_]^2+/3+^ and [Cu(dmp)_2_]^1+/2+^. Republished with permission of Royal Society of Chemistry, from ref. [20], Copyright (2018).

With these limitations in mind, alternative redox shuttles with promising characteristics have been developed, which have reduced the mismatch in the oxidation states between the dye and the redox couples as the one-electron redox mediators (e.g., copper, cobalt, nickel metal transition complexes). These have also provided wider windows between the Fermi level of the semiconducting metal oxides (e.g., TiO_2_) and their own redox potentials to achieve high open circuit voltages, as presented in Figure 7 [20,154].

The one-electron redox mediator can be used to increase the photovoltage, as cobalt based complexes, by tuning the coordination sphere of the complexes [168]. Another advantage is the simpler mechanism for the dye regeneration for the one-electron transfer redox mediators as compared to the two-electron transfer. In the case of a cobalt complex, the dye regeneration reaction consists of the electron transfer from the Co(II) complex to the dye in a oxidized form producing a Co(III) complex (Equation (12)) [169,170].

(12) $$\mathrm{Co}\left(\mathrm{II}\right)+S^{+}\;\underset{\;}{\to }\mathrm{Co}\left(\mathrm{III}\right)+S^{\;}$$

The electron transfer rate for cobalt complexes are known to be slow due to the spin change (d^7^ Co(II) high spin and d^6^ Co(III) low spin), increasing the internal reorganization energy [168,169,170]. Additionally, the electron transfer rate is also dependent on the structure of the dye and redox mediator, which should affect the reorganizational energy and the electronic coupling [168,171]. In the case of the [Cu(dmp)_2_]^2+/1+^ complex, the internal reorganizational energies are lower than cobalt complexes, due to the coordination geometry of the complex remain the same during the change of the oxidation state (Cu(I) to Cu(II)) [172]. The driving force for dye regeneration can be obtained while considering the difference between the formal potential of the redox mediator and the oxidation potential of the dye in the ground state [171]. The driving force of the cobalt complex for dye regeneration is higher when compared with the copper complexes [172,173].

Such alternative mediator couples should ideally also demonstrate enhanced physical and chemical properties relative to the conventional tri-iodide couple, such as better solubility, substantial optical transparency at concentrations allowing for optimal conductivity, and high thermal stability. They should also display non-corrosiveness towards other components of the solar cell [30,42,47,154]. The development of alternative redox mediators is a hot topic for further improvements in DSSC technology.

##### Cobalt-Based Mediators

One of the most commonly investigated alternative redox shuttles for high performance DSSCs, apart from the iodide/triiodide mediator, is cobalt tris-bipyridine ([Co(bpy)_3_]^2+/3+^). However, in initial trials, the charge-transfer dynamics in this cobalt complex yielded a very low PCE of no more than 2.2% under full sun illumination while employing Z316 an organic sensitizer [174]. This low efficiency was later attributed to the naturally occurring fast recombination process of the conduction band electrons evident in a combination of TiO_2_ with Co^3+^ species. 

Generally, cobalt redox mediators have characteristics, such as nonvolatility, non-corrosiveness, light-coloration for less light absorption, and, through the alteration of ligands, they achieve a variable electric potential window (0.3−0.9 V) [16,175,176]. Nevertheless, because of their bulky size and high viscosity, these redox couple-based electrolytes initially raised concern related to their mass-transport limitation and recombination losses [16]. 

Numerous efforts were made to overcome mass-transport and recombination losses [177,178,179], which revealed that, for a traditional mesoporous TiO_2_ layer, the diffusion process for Co^3+^ (dtb-bpy) was intrinsically slower than that of the triiodide ions in the electrolyte solution [177]. Recombination kinetics involving the electron lifetimes in different cobalt redox species were found to be low in [Co(dtb-bpy)_3_]^2+/3+^ slightly lower for [Co(dm-bpy)_3_]^2+/3+^ and the lowest in [Co(bpy)_3_]^2+/3+^. However, their recombination rate constants decreased in the opposite sequence, whereby [Co(bpy)_3_]^2+/3+^ demonstrated the smallest constant [Co(dm-bpy)_3_]^2+/3+^ and [Co(dtb-bpy)_3_]^2+/3+^ the highest constant. These results indicate that the structure of the cobalt electrolytes and the porosity of the TiO_2_ films are of paramount importance in defining the performance of the DSSCs [178,179].

In 2011, the cobalt complexes received tremendous attention for their role in significantly boosting the solar-to-electrical conversion efficiency of DSSCs to >12% under full sun illumination. This improved efficiency was achieved by employing a [Co(bpy)_3_]^2+/3+^ redox-based electrolyte, in conjunction with a donor−p−bridge−acceptor zinc porphyrin sensitizer YD2-o-C8 [16]. These efforts were further advanced, while achieving a conversion efficiency of 13%, by using a Co^2+/3+^ redox shuttle, along with a porphyrin dye (SM315), under full sunlight illumination [17]. 

**Figure 8:**
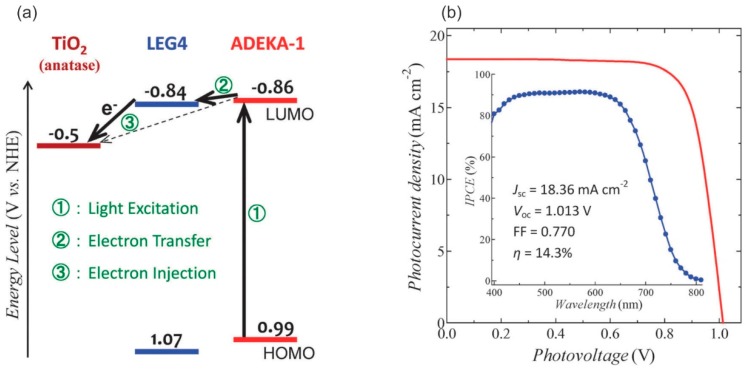
(**a**) Energy level diagram for the LEG4 and ADEKA-1 dyes and (**b**) I–V curve of the co-sensitized DSSC. Republished with permission of Royal Society of Chemistry, from ref. [15], Copyright (2015).

Recently, integrating a [Co(phen)_3_]^2+/3+^ redox couple resulted in an efficiency of 14.3% at AM 1.5 full sunlight irradiation with an alkoxysilyl-anchor dye ADEKA-1 co-sensitized dye with a carboxy-anchor based organic dye LEG4 [15]. This is the highest known PCE that has been reported for DSSCs to date. The combination of co-sensitization in conjunction with a [Co(phen)_3_]^2+/3+^ redox shuttle produced a considerably high photovoltage of >1.013 V and it also displayed a remarkable photocurrent density of 18.36 mA cm^−2^ (Figure 8) [15]. Hence, cobalt redox shuttles are currently considered as the most efficient mediators for DSSCs. Recent work has proved that smart optimization of their redox potentials, combined with the tunability of the sensitizing dyes, makes DSSCs with very high conversion efficiency possible. 

Interestingly, another recent strategy involves adding small organic additives, such as tris(4-methoxyphenyl)amine (TPAA) or (2,2,6,6-tetramethylpiperidin-1-yl)oxyl (TEMPO), to electrolytes, to aid the main redox mediator via an electrode transfer cascade. This has also worked well for cobalt complex mediators [180,181,182]. DSSCs employing this technique have reached PCEs of up to 9.1% under full sun irradiance, especially in conjunction with the organic dye LEG4 [181,182]. 

A hexadentate cobalt complex with a hexapyridyl ligand (6,6′-bis(1,1-di(pyridin-2-yl)ethyl)-2,2′-bipyridine, bpyPY4) was recently shown to outperform the conventional [Co(bpy)_3_]^2+/3+^ in terms of both PV performance and stability [182,183]. Similar results have been reported utilizing a hemicage structured cobalt mediator [Co(ttb)]^2+/3+^ with a pre-organized hexadentate ligand 5,5′’,5′’’’-((2,4,6-triethyl benzene-1,3,5-triyl) tris(ethane-2,1-diyl)) tri2,2′-bipyridine (ttb) [182,184]. DSSCs constructed with these complexes were found to have overall efficiencies that were comparable to the prototypical Co-bpy redox mediator, however they clearly outperformed the Co-bpy redox shuttle in terms of stability under full sun irradiance [182,184]. 

##### Copper-Based Mediators

Similar to cobalt redox shuttles, copper-based mediators also offer numerous attractive characteristics, such as the efficient regeneration of dyes at extremely small driving force potentials, the ability to attain high photovoltages of around 1.0 V without compromising photocurrent densities, and slower recombination rates. Their recombination rates have recently been tested with several organic dyes, which not only produced high performance DSSCs under full sun illumination, but also contributed to the efficient DSSC operation at low light intensities [19,172,185].

**Figure 9:**
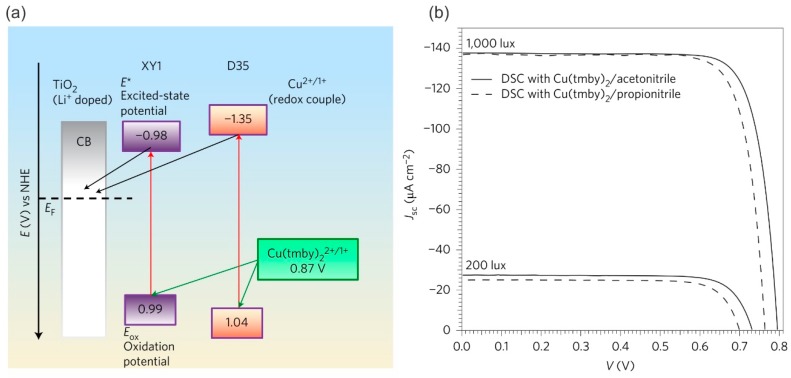
(**a**) Energy level diagram for the XY1 and D35 dyes with the [Cu(tmby)_2_]^1+/2+^ redox couple and (**b**) I–V curve of the co-sensitized DSSC measured under indoor-light conditions. Reprinted with permission from Springer Nature, ref. [19], Copyright (2017).

Recent ground-breaking work has demonstrated that electrolytes that contain Cu mediators perform more efficiently under low light intensities than other types of thin film solar cells [19]. This was achieved by co-sensitization (combining two rationally formulated sensitizers), of D35 and XY1 dyes, and applying the copper complex [Cu(tmby)_2_]^1+/2+^ (tmby = 4,4′,6,6′-tetramethyl-2,2′-bipyridine) as a redox shuttle. This combination not only enabled a substantial open-circuit photovoltage of around 1.1 V, but it also achieved an incident photon-to-electron conversion efficiency exceeding 90% for the generated photocurrent (range 400 to 650 nm). Impressive power outputs of 15.6 and 88.5 μW cm^−2^ were achieved at low light intensities of 200 and 1000 lux, respectively (Figure 9). The entire configuration resulted in a solar-to-electrical PCE of 28.9%, which completely outperformed existing conventional GaAs thin-film PV technology under low light intensity conditions [19].

The same co-workers have also achieved 11.3% solar-to-electrical energy conversion under 100 mW cm^−2^ AM 1.5 G light by using a similar setup with a Cu-based redox mediator and co-sensitized dyes [19]. This exceeded the earlier 8.3% conversion efficiency employing copper^(1+/2+)^ bis(2,9-dimethyl-1,10-phenanthroline) ([Cu-(dmp)_2_]^1+/2+^) as the redox mediator in conjunction with a LEG4 sensitizer [186].

Furthermore, the same group was able to improve overall PCE by introducing two new copper bipyridyl complexes, [Cu(dmby)_2_]^1+/2+^ (dmby = 6,6′-dimethyl-2,2′-bipyridine) (0.97 V vs. SHE) and [Cu(tmby)_2_]^1+/2+^ (tmby = 4,4′,6,6′-tetramethyl-2,2′-bipyridine) (0.87 V vs. SHE), as the redox mediators that were employed alongside a new broad spectrum organic dye Y123. This new configuration resulted in a PCE of 10.3% for [Cu(tmby)_2_]^1+/2+^ and a PCE of 10.0% for [Cu(dmby)_2_]^1+/2+^, both under full sun irradiation [172]. The previously reported mediator ([Cu-(dmp)_2_]^1+/2+^) [186] was also tested with Y123 and, surprisingly, it also yielded a high PCE of 10.3% under full sun irradiation [172]. 

Hence, Cu mediators have shown immense potential in recent studies despite being a new entrant in DSSC technology and are gaining attention as candidates that are able to further boost solar-to-electrical conversion efficiency of the next generation of DSSCs. 

##### Ferrocene-Based Mediators

The ferrocenium/ferrocene redox couple possesses unique characteristics, including extraordinary electrochemical properties, such as fast dye regeneration, and small driving force potentials providing sufficient *V_OC_* and good photocurrents. These properties and their abundant availability in the earth’s crust make them one of the most favourable mediators for further research [154,187,188]. 

One principal disadvantage of the ferrocenium/ferrocene couple is high recombination, following the photoinjection of the electron into the TiO_2_ layer [187,188]. This has led to studies that focus on understanding and controlling these interfacial recombination processes. In a profound study, recombination occurring between the SnO_2_ layer and the electrolyte solution interface was differentiated from that occurring between the nano-porous TiO_2_ layers and the electrolyte solution interface [187]. Recombination was more dominant in the former for dark measurements, whereas recombination was more common at the latter interface for illuminated measurements. 

Two methods were tested to address these limitations. The first involved electropolymerization of an insulating film of poly(phenylene oxide-co-2-allylphenylene oxide) deposited on top of those parts of the SnO_2_ surface, exposed to the solvent. The second involved depositing an insulating PMS (polymethylsiloxane) film on the uncovered sensitized surfaces of both the TiO_2_ and SnO_2_ by chemical vapor deposition of reactive methyltrichlorosilane vapor. Both of these reactions were detrimental to the interfacial charge recombination rate by forming an electrochemical barrier for the recombination back electrons. Nevertheless, these passivation layers still allowed free passage to forward electrons of the fast Fc^+^ redox mediator [187]. The electrodeposited blocking layer of cross-linked PPO on the TiO_2_-coated SnO_2_ surface was also produced before dye-sensitization, which resulted in the development and adherence of PPO on the dye-adsorbed TiO_2_ layer. This treatment decreased the recombination rates of the photoinjected electrons originating from the mediator, thereby considerably improving the overall efficiency of the DSSC [187].

The insulating PMS film on the photoanode surface effectively impedes the titania-electrolyte electron-transfer processes. The overall efficiencies that were achieved were 0.36% and 0.51% after two rounds of silane application on photoanodes that were sensitized with N719 and Ru(bpy)_2_(dcbpy) dyes, respectively, under full sun illumination [188]. It is noteworthy that no PV effect was observed in DSSCs without this passivation treatment, because almost all of the photogenerated charge carriers instantly recombined before the measurements could be performed [188]. 

Ultra-thin coatings of alumina have also been fabricated via atomic layer deposition over TiO_2_, or magnetron sputtering to produce compact TiO_2_ layers was also tested with these shuttles. These modified electrodes allow for optimal utilization of the Fc^0/1+^-based electrolyte solution and demonstrated minor improvements in current densities’, and confirmed that an additive passivating layer may enhance the overall PV operation [189,190]. 

Although these treatments have a positive impact on current densities, their efficiencies remained low (*η* < 0.4%). The starting efficiencies of these devices were in the range of 0.1–0.2%, and therefore doubling the efficiency did not have a significant effect [187,188,189,190].

In contrast, a far superior PCE of up to 7.5% under full sun illumination has been demonstrated in DSSCs that integrate a ferrocene/ferrocenium Fc^0/1+^ single-electron based redox couple with a metal free organic sensitizer (i.e., Carbz-PAHTDTT). This clearly exceeded the efficiencies of I^–^/I_3_^–^ electrolyte-based DSSCs [191]. Hence, ferrocene-based mediators show promise as compatible match with organic sensitizers in future DSSC applications. 

Basic alkylation and halogenation of the cyclopentadienyl ring can also formulate a series of ferrocene derivatives, resulting in other redox species, such as Br_2_Fc^0/+^, BrFc^0/+^, EtFc^0/+^, Et_2_Fc^0/+^, and Me_10_Fc^0/+^. The redox potentials of these derived species are considerably broad in range (0.09–0.94 V vs. NHE) [192]. The solar conversion efficiencies demonstrated by these new derived species, Fc, Et_2_Fc, and EtFc, varied from 4.3–5.2% under full sun. BrFc and Br_2_Fc displayed lower energy conversion efficiencies in comparison to EtFc, Et_2_Fc, and Fc amongst the new derived species, regardless of their higher redox potentials. This is explained by the fact that the latter species promoted faster dye regeneration, with effective driving forces in the range of 35–46 kJ mol^−1^ [192]. 

Further improvements in the efficiency of DSSCs employing Fc^0/+1^ electrolytes would require the ferrocene derivative species to adequately complement higher performing dyes with suitable energy levels. This should not exceed ΔE beyond 0.36 V, as has been observed for the cobalt-based redox shuttles [192]. Therefore, the experimental results suggest that variants of ferrocene derivatives, and especially the halogenated and alkylated ones, should be applied in DSSCs with organic dyes and/or panchromatic dyes. 

##### Nickel-Based Mediators

Nickel couples of Ni (III)/(IV) bis(dicarbollides) have also been tested and established as an alternative mediator species for electrolytes in DSSCs, similarly to ferrocene complexes. This acts as a fast, single-electron outer sphere redox couple with non-corrosive properties [193,194]. The benefits of the Ni (III)/(IV) bis(dicarbollides) shuttle include striking electron transfer rates, rapid dye regeneration, and swift mass transport, especially when compared to the Fc/Fc^+^ shuttles [154]. Improvement in the efficiency (1.5% under AM 1.5 illumination) of the fabricated DSSC was achieved through TiO_2_ surface passivation with conformal deposition of Al_2_O_3_ via atomic layer deposition (ALD). In comparisons to redox species at the same concentration (0.030 M), the regeneration of the Ni couple-based electrolyte was found to be more efficient than that of the conventional iodide electrolyte, and the rate of recombination was 1000 times lower than that with a ferrocene electrolyte. This improvement was believed to be a result of an activation barrier for the reduction of Ni^4+^ to Ni^3+^ [193].

Furthermore, the redox potentials of nickel-based complexes have also been modified, like other redox mediators. This has been achieved by the functionalization of the Ni (III)/(IV) bis(dicarbollides) complex with electron-donating and electron-withdrawing groups [194]. The electron withdrawing groups that were associated with the complex generally caused the redox potential to be more positive, leading to higher *V_OC_* values. PCEs of 0.7–2.0% under full sun AM 1.5G illumination were achieved with these Ni^3+/4+^ shuttles [194].

In comparison to DSSCs comprising photoanodes that were purely fabricated from TiO_2_ nanoparticles, more than 100% improvement in photocurrent densities has furthermore been achieved by employing high surface area photoanodes. These photoanodes, comprised of silica aerogels coated with ALD over TiO_2_ layers, in conjunction with Ni (III)/(IV) bis(dicarbollides) as the Ni-based redox shuttle, demonstrated improved performance in terms of efficient photocurrent generation [195]. The improvement in photocurrent resulted from many factors, including enhanced electron transference, curtailed recombination losses at the TiO_2_/electrolyte boundary layer, and amplified light scattering in the aerogel films [195]. As a result, DSSCs with aerogel PEs exhibited considerably higher photocurrent densities (6.3 mA/cm^2^) and PCEs (2.1%) than the DSSCs that utilized nanoparticulate PEs, in combination with the Ni shuttle system [195].

Long-term device stability has rarely been reported or investigated although the main characteristics and efficiencies of these alternative redox shuttles have been studied, thus raising concern for their reliable long-term operation in DSSCs. Future research should investigate the long-term device stability of DSSC-based PV systems integrating these alternative redox shuttles. In addition, studies of the corrosive behavior of these alternative redox shuttles towards metal substrates are needed [105]. Table 4 summarizes the best efficiencies that have been reported so far with electrolytes based on alternative redox mediators.

### 4.2. Gel Electrolytes (Quasi-Solid State Electrolytes)

Leakage and sealing issues impacting on long-term device stability remain the key barrier to their successful commercialization although DSSCs with liquid electrolytes show the best known solar-to-electrical conversion efficiencies [112,196]. Volatile liquid electrolytes can be replaced with gel electrolytes to overcome these limitations [37,197,198].

Gel electrolytes (or quasi-solid electrolytes) contain a polymeric matrix that acts as a framework for the solvent and inorganic salts that are used as additives [199,200]. The polymer matrix used to create a three-dimensional network can be either inert or coordinating [197]. In inert gel electrolytes (e.g., poly(vinylidene fluoride) (PVDF), poly(acrylonitrile) (PAN)) the cations and anions can move freely in the solvent (liquid phase). Gel electrolytes with coordinating polymers, such as poly(ethylene oxide) (PEO), present a binary phase (liquid and solid), where the ionic transportation occurs [197,201]. The gel system is not considered either liquid or solid, but, as a hybrid structure with the diffusive transport properties of a liquid and cohesive properties of a solid [200]. 

In general, gel electrolytes are easy to prepare and they present very good ionic conductivity (i.e., 10^−5^–10^−3^ S cm^−1^), low solvent volatility, and good chemical and mechanical stability [200,202]. They have excellent properties for filling the TiO_2_ pores and they provide an excellent contact between the CE and PE [203,204]. Another attractive characteristic of gel electrolytes is their high viscosity, which makes them compatible for roll-to-roll deposition methods [205,206,207,208,209], contributing to cheaper manufacture of DSSC modules [210].

Gel electrolytes have been extensively studied over the last 20 years for DSSCs with iodide redox couples, and more recently with cobalt redox shuttles. These gel electrolytes can be classified into three categories based on their preparation [36]:

(i) A liquid electrolyte can be solidified by adding a polymer matrix, which acts as a gelator. The resulting gel polymer electrolytes can be further classified according their formation mechanism, as either thermoplastic (for physical cross-linking) or thermosetting (for chemical cross-linking) [211,212,213].
(ii) A liquid electrolyte containing a polymeric matrix can be solidified by inorganic or organic gelators (e.g., SiO_2_, TiO_2_, nano-clay powder, carbon-based materials), which results in a composite polymer electrolyte [214,215,216,217,218].
(iii) A quasi-solid ionic liquid electrolyte can be prepared by adding a gelator, such as a polymer matrix or inorganic nanoparticles, to the ionic liquid electrolyte [219,220,221].

#### 4.2.1. Gel Polymer Electrolytes

Gel polymer electrolytes that are prepared by chemical or physical cross-linking process are called “thermosetting polymer electrolytes—TSPE” [212] or “thermoplastic polymer electrolytes—TPPE” [213], respectively. Chemical cross-linking occurs when covalent bonding of polymer chains is created by chemical reactions, leading to the formation of thermo-irreversible gels. On the other hand, gels that formed by physical cross-linking arise from weak interactions between the polymeric matrix and solvents, such as hydrogen bonds, Van der Waals interactions, electrostatic interactions, and others. The gel electrolyte that was obtained via physical cross-linking is termed an entanglement network, and it is thermo-reversible [36,37,198].

Numerous polymer hosts can be used in DSSCs. Table 5 and Table 6 show some of physical properties of the most common polymers and solvents applied to prepare gel electrolytes for DSSCs.

##### Thermoplastic Polymer Electrolytes (TPPE)

**Figure 10:**
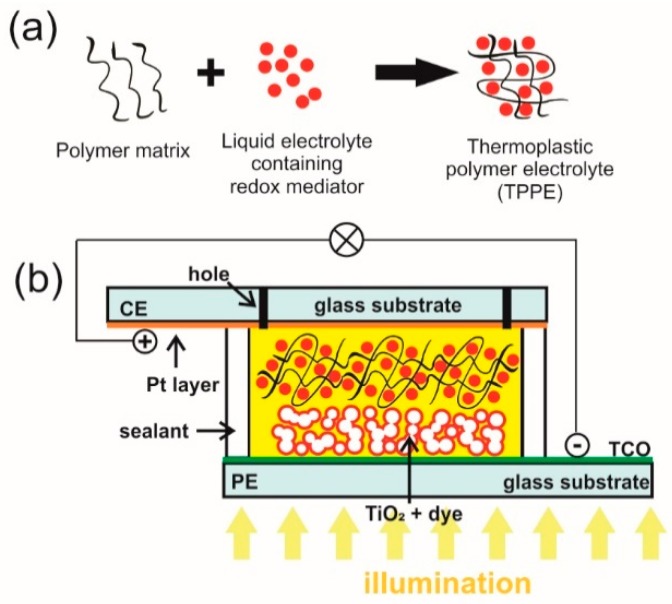
Schematic illustrations of (**a**) swelling the polymer network with liquid electrolyte containig the redox mediator such as presented in ref [212] and (**b**) Schemcatic illustration of a DSSC containing the gel electrolyte such as described in ref [224].

The main characteristic of this kind of electrolytes is their reversible temperature-controlled transition from solution to gel state [37]. TPPEs can be prepared by mixing a polymeric matrix or oligomer with a liquid electrolyte, which already contains an organic solvent, a redox couple, and other additives [37,212,224,225]. The polymer or oligomer are termed gelators and they act as a framework to form a viscous homogeneous system with the liquid electrolyte that is trapped inside the polymer host structure [196,198]. The organic solvent provides a medium for the migration of ionic salts through the free volume or micropores of the polymer matrix [198]. Figure 10 illustrates the gel structure that contains the trapped liquid electrolyte and its application in DSSCs. In some cases, the organic solvent can act as a plasticizer, reducing the degree of crystallinity and changing the glass transition (Tg) of the polymer. In this case, the organic solvent and the polymer matrix should contain coordinating/solvating atoms (e.g., γ-butyrolactone, phthalic acid esters, PVC, PEO). The plasticizer is added to increase the flexibility of the polymeric chains by introducing some degree of disorder in the crystalline phase of the polymer. As a result, the polymer-polymer chain interactions are reduced and the segmental mobility of the polymeric chain increases [196,197,198].

Cao et al. first introduced TPPEs DSSCs [226]. A gel polymer electrolyte with a mixture of poly(acrylonitrile) (PAN) polymer, ethylene carbonate (EC), propylene carbonate (PC), and ACN as the solvent and NaI and I_2_ was prepared and revealed a comparable open circuit voltage (*V_OC_*) and *FF* to those that were achieved with liquid electrolytes. DSSCs containing these gels exhibited 3–5% solar-to-electrical conversion efficiencies when measured under full sunlight illumination. 

The predominant polymer matrix that was used to prepare polymer gel electrolytes for DSSCs is based on PEO. Initially, the PEO copolymers were used to prepare polymer electrolytes in a solid state (without solvent) due their ability to efficiently dissolve inorganic salts. In this case, a Lewis type acid-base interaction occurs between the electron donor pairs in the oxygen atoms of the polymer structure and the alkali metal cations [196,198].

The PEO polymers have been used to produce solid state polymer electrolytes for DSSCs [227]. In the first such study, poly(o-methoxyaniline) was used as the sensitizer and a copolymer of poly(epichlorohydrin-co-ethylene oxide) containing NaI/I_2_ as electrolyte, reaching an efficiency value of 1.3% with irradiation at 410 nm. In another study, devices with 0.22% efficiency under 120 mW cm^−2^ illumination were prepared while using the same copolymer and a complex of ruthenium as the sensitizer [228]. However, solid polymer electrolytes usually present low ionic conductivities, in the order of 10^−8^–10^−5^ S cm^−1^ and poor contact between the electrodes, limiting cell performance [203,229]. For this reason, PEO-based polymers have mostly been used as gel electrolytes in DSSCs, usually with I^−^/I_3_^−^ redox mediators, or more recently with cobalt-based redox mediators [230,231,232,233].

Shi et al. prepared a polymer gel electrolyte while using a PEO polymer matrix (Mw = 2 × 10^6^ g mol^−1^) to gel the liquid electrolyte with a weight ratio ranging from 2.5 to 15 wt % [234]. They observed PEO to improve the mobility of Li^+^, which decreased internal cell resistance. The optimized DSSCs reached 6.12% energy conversion efficiency under full sun illumination using 10 wt % of PEO. More importantly, the stability of these fabricated DSSCs was improved with the increase of polymer amount [234].

Recently, urea has been used as a plasticizer for PEO to prepare polymer gel electrolytes with an I^−^/I_3_^−^ redox couple [235]. The presence of 4% urea increased the ionic conductivity (σ = 42.8 mS cm^−1^) and the tri-iodide diffusion coefficient (D = 2.06 × 10^−6^ cm^2^ s^−1^). An impressive 6.82% solar-to electrical conversion efficiency was achieved under 85 mW cm^−2^ light intensity. 

Moreover, a TTPE for DSSCs can be prepared with poly(ethylene glycol) (PEG) (40%) as the polymer matrix, propylene carbonate (PC) (60%) as the solvent and I^−^/I_3_^−^ as the redox couple [212]. The ionic conductivity of the electrolyte was 2.61 mS cm^−2^ and the energy conversion efficiency was 7.22%, as compared with liquid-based devices (7.60%) under 100 mW cm^−2^ illumination. The long-term stability of these DSSCs was also greatly improved.

The PVDF-HFP copolymer is another widely used polymer matrix in the preparation of TTPEs [236,237,238,239]. This polymer contains two monomers: a symmetrical and crystalline VDF (vinylidene fluoride) and the asymmetrical amorphous HFP (hexafluoropropylene), which contribute to the high ionic conductivity and good mechanical strength of the copolymer [202].

The PVDF-HFP was used to prepare a polymer gel electrolyte for bifacial DSSCs (as discussed in Section 3) [240]. An energy conversion efficiency of 10.37% was obtained with an I^−^/I_3_^−^ redox couple and a liquid electrolyte containing 6 wt % of PVDF-HFP, which was comparable with that obtained with a liquid-based DSSC (η = 9.89%), both being measured under 100 mW cm^−2^ irradiation. A long-term stability test showed that the liquid-based cells survived for only 200 h, while the gel-based devices were unchanged after 1000 h.

A printable electrolyte was prepared by using 9 wt % of a polymer blend of PEO and PVDF to solidify an iodide-based liquid electrolyte containing a 3-methoxypropionitrile organic solvent [205]. The devices reached 8.32% efficiency, which was very similar to that of the liquid cells (η = 8.34%) under 100 mW cm^−2^ irradiation. These DSSCs showed high long-term stability at 60 °C thermal stress under dark conditions.

For cobalt-based mediators, the most common polymer matrix that was used for TTPE electrolytes is PVDF-based, mainly due its high thermal stability [241,242,243]. TPPE electrolytes with cobalt mediators were investigated for the first time by Xiang et al. [244]. The authors studied the influence of varying the amount of the PVDF-HFP copolymer from 0 to 10 wt % against the performance of the dye solar cells, which were fabricated with MK-2 organic dye loaded PEs [244]. Devices that were prepared with 4 wt % of a PVDF-HFP-based electrolyte exhibited 8.7% efficiency under 100 mW cm^−2^ illumination, which remained stable for a period 700 h under continuous full sun illumination [244]. However, the performance of the devices was reduced with an electrolyte containing 10 wt % of the polymer, which suggested a drop in the diffusion rate of the redox mediator through the electrolyte medium [244].

Another interesting application of PVDF-HFP in preparing TTPEs was made by using the reactive TEMPO (2,2,6,6-tetramethyl-piperidin N-oxyl) radical/cation as a redox couple with PVDF-HFP to form a polymer gel electrolyte. The DSSCs were sensitized with an indoline dye (MD-153) and they reached an efficiency of 10.1% under 100 mW cm^−2^ illumination. The TEMPO radical exhibited a rapid self-exchange activity and redox activity, resulting in fast charge transport [245].

##### Thermosetting Polymer Electrolytes (TSPE)

As mentioned before, TSPEs are prepared by chemical cross-linking and they are not reversible [246]. One of the main drawbacks of using thermoplastic polymer electrolytes is their penetration of the TiO_2_ photoanode pores due to their high viscosity. This problem can be addressed with TSPEs, in which the polymerization reaction is only initiated after monomer infiltration into the TiO_2_ pores [247]. TSPEs have greater chemical and thermal stability when compared with TTPEs, although their ionic conductivity is lower [36]. 

**Figure 11:**
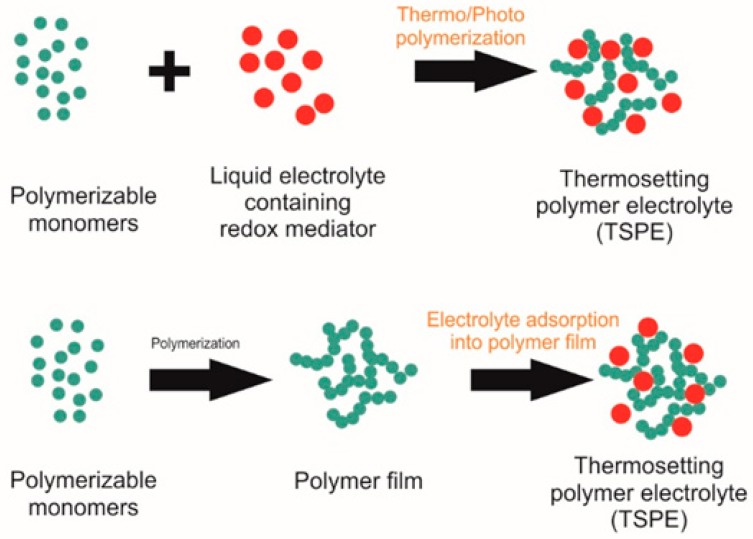
Schematic illustrations of Thermosetting Polymer Electrolytes (TSPE) preparation methods: thermo- and photopolymerization and electrolyte adsorption [248,249,250,251,252,253].

Figure 11 depicts the different methods available to prepare TSPE electrolytes, such as thermo- [248,249,250] or photo- [251,252,253] in situ polymerization and the liquid electrolyte adsorption method [254,255]. In short, the monomer that is presented into liquid electrolyte is polymerized with the aid of an initiator and the presence of light or heat. For the adsorption method, the polymerization occurs first and the electrolyte is then adsorbed in the polymeric matrix.

The first example of a TSPE that was prepared by light-induced in situ polymerization used a α-methacryloyl-ω-methocyocta (oxythylene) polymer matrix, which was polymerized at the TiO_2_ porous film, followed by the immersion of the resulting film into a liquid electrolyte [256]. The devices presented 2.62% energy conversion efficiency under full sunlight illumination.

In 2005, Wang et al. produced a TSPE by using latent chemically cross-linking gel electrolyte precursors, which infiltrated into the TiO_2_ photoanode pores followed by heating at 80 °C to solidify the electrolyte [247]. The precursors comprise polypyridyl-pendant poly(amidoamine) (PAMAM) dendritic derivatives (PPDD) and a dysfunctional halogen derivative (DHD) of PEO with iodine groups on the chains ends. The resulting quasi-solid devices presented 7.72% efficiency under full sunlight illumination, with an I^−^/I_3_^−^ redox couple.

Dong et al. prepared a gel copolymer that was used to absorb a liquid electrolyte by the oligomerization of PEO-segmented diamine and an 4,4′-oxydiphtalic anhydride at high temperature to form a gel that is based on amide-imide crosslinking [257]. Devices that were prepared using 76.8% liquid electrolyte and an I^−^/I_3_^−^ redox couple had an energy conversion efficiency of 9.48% under full sunlight illumination. This achievement was related to high ionic mobility in the gel channels and the good contact of the electrolyte with the photoanode and CE.

In a distinct approach, Yang et al. prepared an ultra-thin porous PVDF-HFP membrane by the phase inversion method on the photoanode. The organic liquid electrolyte was infiltrated into the membrane to form an in situ ultra-thin porous membrane electrolyte. Devices with this membrane electrolyte reached an energy conversion efficiency of 8.35% under full sunlight illumination [258].

On the other hand, Park et al. used the precursors methyl methacrylate (MMA) and 1,6-hexanediol diacrylate (HDDA) to prepare a tunable nano-porous cross-linked polymer film on the TiO_2_ articles, which selectively transports ions, depending on their size [259]. The energy conversion efficiency of their devices was 10.6% under full sunlight illumination and this was related to dark current suppression. The devices revealed excellent long-term stability for almost 600 h. 

The first TSPE electrolyte with cobalt mediator has recently been developed by in situ polymerization by UV radiation of a dimethylmethacrylate oligomer (BEMA) with poly(ethylene glycol) methyl ether methacrylate (PEGMA) [260]. The oligomer acts as a plasticizer, decreasing the glass transition temperature (Tg) of the resulting polymer, and thus increasing the mobility of the cobalt complex, by virtue of ethoxy groups present on the polymer chain. The performance of the resultant DSSC reached 6.4% under 100 mW cm^−2^, with the LEG 4 organic dye. The stability tests indicate that cells containing this quasi-solid electrolyte exhibited a decay of only 5% relative to their initial efficiency after 1500 h.

#### 4.2.2. Composite Polymer Electrolytes

**Figure 12:**
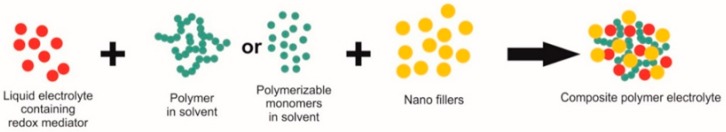
Schematic illustrations of composite polymer electrolyte preparation [261].

Nanoparticles can be added into polymer matrix, producing a composite polymer electrolyte. The nanoparticles inside the polymer electrolyte system helps to reduce the crystallinity of the polymer, as well to create a three-dimensional porous system structure [261]. Subsequently, the redox mediator can easily be diffused through this porous system and, consequently, improves the ionic conductivity as well the photocurrent and efficiency of devices [261,262,263]. In general, the polymer gel electrolytes containing inorganic or organic nanoparticles present improved mechanical, interfacial, and conductive properties. Composite polymer electrolytes can be prepared by the addition of inert fillers, usually inorganic nanomaterials, such as Al_2_O_3_, TiO_2_, ZrO_2_, SiO_2_, or carbon-based nanoparticles, into an electrolyte containing a polymer matrix [203,246,261,262,263]. Figure 12 shows a general schematic of composite polymer electrolyte preparation.

##### Inorganic Nanoparticle-Based Composite Polymer Electrolytes

Croce et al. introduced inorganic nanoparticles to jellify electrolytes for the first time [264]. They used TiO_2_ and Al_2_O_3_ nanoparticles that were mixed with PEO-LiCLO_4_. This resulted in an improvement of ionic conductivity, which reached 10^−4^ S cm^−1^ at 50 °C. Electrolytes that are based on these types of material have some interesting characteristics, including good flexibility, high thermal stability, good interfacial contact between the electrodes, and high ionic conductivity [202]. Moreover, the addition of inorganic nano-fillers into the electrolyte system reduces the crystalline structure of the polymers, which facilitates ion dissociation, and consequently improves the charge transfer through the electrolyte/CE interface [204].

TiO_2_ is the most widely used inorganic nanoparticle in the preparation of composite polymer electrolytes. Chen et al. prepared devices with an energy conversion efficiency around 10% under full sunlight illumination, exceeding that of their liquid counterparts by using a polymer matrix of poly(acrylonitrile-co-vinyl acetate) (PAN-VA) with TiO_2_ nano-filler [265]. The electrolytes were obtained by immersing different compositions of copolymers into the I^−^/I_3_^−^ liquid electrolyte. Improved efficiency was related to the synergistic effects of the polymer matrix and the TiO_2_, which influenced the charge transfer at the CE interface [265].

Wang et al. produced a polymer gel electrolyte while using polyvinyl(acetate-co-methyl methacrylate) [P(VA-co-MMA)] as the polymer matrix with ACN or 3-methoxypropionitrile (MPN) as the organic solvent and with an I^−^/I_3_^−^ redox couple [209]. The addition of a TiO_2_ nanofiller (5 wt %) improved the ionic conductivity and energy conversion efficiency from 9.10% to 9.40% for the ACN-based electrolyte and from 8.61% to 8.98% for the MPN-based electrolyte. The devices showed good long-term stability under 1000 h of light exposure. All of the measurements were made under 100 mW cm^−2^ illumination.

A PEO-based composite polymer electrolyte, as prepared by Seo et al., was obtained by adding 5 wt % of TiO_2_ nanoparticles [206]. Solidification of the liquid electrolyte containing an I^−^/I_3_^−^ redox couple with the polymeric matrix, gave rise to improvements in *V_OC_* and the introduction of nanofillers increased the redox ion transport. The energy conversion efficiency of the devices reached 9.2% at 100 mW cm^−2^ illumination.

A polymer blend can also be used to prepare composite polymer electrolytes. Zebardastan et al. prepared a gel electrolyte with PVDF-HFP and PEO with SiO_2_ as the nanofiller and an I^−^/I_3_^−^ redox couple [266]. The highest ionic conductivity obtained was 8.84 mS cm^−1^, with 13 wt % of SiO_2_ giving rise to an energy conversion efficiency of 9.44%. Liu et al. prepared a printable electrolyte that was composed of a polymer blend of PEO and PVDF containing 4 wt % of TiO_2_ nanofillers, an I^−^/I_3_^−^ redox couple, and MPN as the solvent [205]. Their quasi-solid devices reached 8.91% efficiency under 100 mW cm^−2^ illumination, which was higher than that of liquid cells (η = 8.34%). 

In the literature, the classification of composite polymer electrolytes is basically related to the electrolyte containing polymer matrix, nanoparticle filler used for solidification, and the I^−^/I_3_^−^ redox couple. However, there are some cases where nanoparticle-based fillers alone have been used to solidify the liquid electrolyte medium. For example, Stergiopoulos et al. prepared, for the first time, a quasi-solid electrolyte based on cobalt complex for DSSCs [267]. They used the SiO_2_ nanoparticles to solidify a liquid electrolyte containing [Co(bpy)_3_]^2+/3+^(2,2’-bipyridine), and TBP and LiClO_4_ as additives, both dissolved in 3-methoxypropionitrile (MPN). Doing so resulted in energy conversion values of 2.6% under 100 mW cm^−2^, for a DSSC with D35 organic dye. 

##### Carbon-Based Composite Polymer Electrolytes

Carbon-based materials, such as SWCNTs, MWCNTs (multi walled carbon nanotubes), carbon nanoparticles, or graphene, can also act as gelators to form composite polymer electrolytes [268,269]. The advantage of applying nanosized carbon materials include their good conductivity and the increase in the surface area suitable for electron-transfer [270].

Mohan et al. prepared a composite polymer electrolyte that was based on a poly(acrylonitrile) (PAN) polymeric matrix as the gelling agent and activated carbon (AC) or SiO_2_ as the inorganic nanofiller [271]. They found that the PAN/AC composite polymer electrolyte exhibited better efficiency (η = 8.42%) and electrical conductivity (8.6 mS cm^−1^) than PAN/SiO_2_ (η = 7.51% and 1.32 mS cm^−1^) under full illumination. These results are related to the lower internal charge transfer resistance, higher catalytic behavior, and a more porous morphology that is favourable to ionic transportation of the PAN/AC composite polymer electrolyte [271].

A carbon nanotube (MWCNT) suspension with poly(oxyethylene)-segmented oligo(amide-imide) (POEM) as the dispersant was prepared in aqueous media by Wang et al. [272]. This mixed composition paste was added to a gel electrolyte containing 5 wt % of PVDF-HFP and an iodide/triiodide redox mediator in MPN. Devices with 0.25 wt % of MWCNT/POEM had 6.86% energy conversion efficiency versus 4.63% for the devices without carbon nanotubes under 100 mW cm^−2^ illumination. The enhanced device performance was due to the formation of a homogeneous system with PVDF-HFP and the MWCNT/POEM dispersion. The aromatic and amide-imide functionalities in the POEM chain interacted with the MWCNTs and chelated Li^+^ ions, increasing the mobility of the I^−^ ions in the electrolyte [272].

Gun et al. applied a low concentration of graphene oxide (GO) to solidify an organic solvent, ACN, containing an I^−^/I_3_^−^ redox couple [273]. Devices with this gel electrolyte (0.4 wt % graphene oxide) presented an energy conversion efficiency of 7.5% versus 6.9% for the devices without GO under 100 mW cm^−2^ illumination.

Zheng optimized the formulation of a quasi-solid electrolyte consisting of a PAA/PEG polymeric matrix containing graphene and I^−^/I_3_^−^ redox couple [274]. The best energy conversion efficiency that was obtained was 9.1% under 100 mW cm^−2^ illumination. The gel electrolyte provides a framework for ion diffusion, whereas the graphene assists in the reduction of the triiodide ions in the three-dimensional framework of the microporous conducting gel electrolyte, and not only at the Pt/electrolyte interface [274].

Recently, Venkatenasan et al. prepared a printable electrolyte that was based on a 9 wt % PEO-PVDF (8/2) polymer blend, a graphene oxide sponge (GOS) as the nanofiller, and an I^−^/I_3_^−^ redox couple [208]. Their quasi-solid devices with 1.5 wt % GOS reached an energy conversion efficiency of 8.78%. The presence of GOS was found to increase the diffusivity and conductivity of the printable electrolyte. A long-term stability test showed that 86% of the initial efficiency of devices was retained after 500 h at 60 °C under dark conditions [208].

#### 4.2.3. Quasi-Solid Ionic Liquid Electrolytes

Ionic liquids are very versatile materials and they can be used to prepare liquid [137,161], quasi-solid [275,276], and even solid electrolytes [277]. Ionic liquids present high thermal stability and they are able to dissolve many kinds of inorganic and organic compounds. They have low flammability and, due their strong electrostatic interactions, are practically non-volatile solvents [221]. These characteristics make ionic liquid suitable as electrolytes for DSSCs with high long-term stability.

Wang et al. solidified an electrolyte that was based on an ionic liquid for the first time with nanoparticles [278]. They mixed SiO_2_ with liquid electrolytes based on MPII (1-methyl-3-propylimidazolium iodide). Devices that are based on this exhibited a conversion efficiency of 7% under 100 mW cm^−2^ illumination. The same group was also the first to integrate an ionic liquid with a gel polymer electrolyte [279]. They combined a PVDF-HFP polymer matrix with MPII to produce an ionic liquid electrolyte in a gel structure, obtaining devices with a conversion efficiency of 5.3% at 100 mW cm^−2^ illumination.

Nanoparticles fillers and polymer matrices can also be combined to obtain quasi-solid ionic liquid electrolytes. Alumina nanoparticles (Al_2_O_3_) were used to obtain an ionic liquid electrolyte in a gel structure by Chi et al. [280]. The authors used Al_2_O_3_ surface modification with an ionic liquid to increase their miscibility with MPII. The quasi-solid electrolyte that was prepared was I_2_-free and the devices pesented an energy conversion efficiency of 7.6% at 100 mW cm^−2^ illumination.

Kang et al. have studied TiO_2_ nanoparticles as fillers in the preparation of composite polymer electrolytes, with poly(ethylene oxide) (PEO) as the framework and the oligomer poly(ethylene glycol) dimethyl ether (PEGDME) [281]. The ionic liquid 1-propyl-3-methylimidazolium iodide (PMImI) (1.20 M) and iodine (0.12 M) were used as mediators. The TiO_2_ nanoparticles improved the energy conversion efficiency (η = 7.2%, at 100 mW cm^−2^ illumination) of these devices, due to the scattering layer behavior of these nanofillers, which also improved the ionic transport [281].

Recently, a highly efficient DSSC (η = 9.61% at 100 mW cm^−2^ illumination) was prepared with phthaloylchitosan/PEO, tetrapropylammonium iodide (TPAI), and 1-butyl-3-methylimidazolium iodide (BMII) to obtain an ionic liquid electrolyte in a gel structure [282]. The phtaloylchitosan was obtained from the chitosan biopolymer and this was blended with a PEO-based polymer to improve the ionic conductivity of the electrolyte.

Nano-clay minerals can also be applied to solidify the ionic liquids and obtain ionic liquid electrolytes in a gel structure for DSSCs. The main advantages of nano-clays are their high chemical stability, swelling capability, ion exchange capacity, and rheological properties [283]. Wang et al. used a synthetic nitrate-hydrotalcite nano-clay to solidify a liquid electrolyte that is composed of 1 M 1-propyl-3-methylimidazolium iodide (PMII), 0.1 M bis(trifluoromethane) sulfonimide lithium salt, 0.1 M iodine, and 0.5 M 1-methylbenzimidazole in an ACN solvent [283]. The energy conversion efficiency reached 9.6% under 100 mW cm^−2^ illumination.

Lee et al. used exfoliated montmorillonite (exMMT) nanoplatelets to produce an ionic liquid electrolyte with a gel framework [284]. The exMMT, with its negative charge, can adsorb 1-methyl-3-propyl-imidazolium cations and jellify the ionic liquid electrolyte. The devices presented an efficiency of 6.58%, which improved to 7.77% under 100 mW cm^−2^ illumination, due to the decay in the resistance of electrolyte medium. Table 7 shows the best efficiencies that were achieved for DSSC employing different gel electrolyte compositions.

### 4.3. Solid State Hole-Transporting Materials

As discussed in the earlier sections, a generic problem in using liquid or quasi solid-state electrolytes in DSSCs is their leakage from the cell channel, which degrades device performance when it is exposed to stressful conditions [14,31,96].

This limitation motivated several research groups to develop a complete solid state DSSC, where the liquid electrolyte was replaced with solid state conducting materials (SSCMs) [31,100,290,291,292,293,294].

These SSCMs retain unique benefits over the electrolytes that were discussed in the previous sections, since they do not comprise solvents at all, which makes them especially suited for large-area DSSC modules. Numerous materials have been investigated and developed to replace the traditional solvent-based (liquid or gel) electrolytes as solid-state conductor materials. Broadly, SSCMs include ionic conductors [290,295,296,297,298,299,300,301,302,303,304], inorganic hole-transport materials [18,31,100,305,306,307,308,309], and organic hole-transport materials [310,311,312,313,314,315,316,317].

#### 4.3.1. Solid State Ionic Conductors

We categorized ionic liquids into RTILs and ionic liquids with higher viscosities and higher melting points earlier in the ionic liquids section. The second category can be termed liquid crystals (LCs). LCs possess the phase properties of both conventional liquids and of solid crystals. Amongst LCs, the discotic liquid crystals (which have either intermediary-phases designed from disc-shaped molecules that are known as discotic mesogens or columnar phases) are widely employed as the charge carrier materials in PV devices [318,319]. Structurally, discotic LCs generally possess an aromatic core that is surrounded by flexible alkyl chains, which provide them with their unique properties [295]. Therefore, discotic LCs can be used as SSCMs in DSSCs. DSSCs utilizing SSCMs are often referred to as solid state DSSCs (ss-DSSCs).

A preliminary study in 2001 of a discotic liquid crystal, hexa-peri-hexabenzocoronene ((HBC-PhC12)), in conjunction with the perylene dye (N,N′-bis(1-ethylpropyl)-3,4,9,10-perylenebis (dicarboximide)), was used to produce a thin film PV device with a large interfacial surface area with vertically segregated perylene and hexabenzocoronene [295]. The PV response in terms of external quantum efficiency was over 34% near 490 nm. The mobilities as high as 0.22 cm^2^ V^−1^ s^−1^ were measured. The *J_SC_* and the *V_OC_* that were measured under the same wavelength with an illumination intensity of 0.47 mW cm^−2^ were −33.5 μA cm^−2^ and 0.69 V, respectively, and the fill factor was up to 40%. The device produced a maximum power efficiency of 1.95% at 490 nm. 

These PV parameters resulted from the moderately efficient photoinduced charge mediation between the LC-based SCCM and the dye, and due to sufficient charge transportation through the vertically segregated perylene and hexabenzocoronene π systems [295]. The study showed that complex structure formulations are possible for new innovative LC-based materials, which are both cost effective and possess high-performance, ideally suited for PV technology is possible, based on simple solution-processing steps [295].

Yamanaka et al. used a novel ionic liquid crystal, 1-dodecyl-3-methylimidazolium iodide, and iodine as a hole-transporting layer for DSSCs [296]. The new ionic liquid crystalline electrolyte (C_12_MImI/I_2_) was fabricated to promote the exchange reaction between I^−^ and I_3_^−^. The concentrations of I^−^ and I_3_^−^ were locally increased, via the introduction of the C_12_MImI/I_2_ liquid crystals with a self-assembled structure of the imidazolium rings in the cations [296]. The new LCs were compared with a similar liquid phase ionic liquid crystal, 1-undecyl-3-methylimidazolium iodide/iodine (C_11_MImI/I_2_), to validate their application at high temperature [296]. C_12_MImI was able to maintain a LC phase up to 80 °C, whereas C_11_MImI lost its liquid crystalline phase at just above 37 °C. The observed diffusion rates for both species revealed the diffusion rate of C_12_MImI/I_2_ (4.2 × 10^−8^ cm^2^ s^−1^) was 1.3 times higher than that of C_11_MImI/I_2_ (3.2 × 10^−8^ cm^2^ s^−1^). At the same time, the viscosity of the new liquid crystals was 2.5 times higher. This led to a higher short circuit current density (*J_SC_*) for C_12_MImI/I_2_ (7 mA cm^−2^) than C_11_MImI/I_2_ (6 mA cm^−2^), and similar *V_OC_* and FF under AM1.5G full sunlight illumination [296].

More recently, two novel double-alkyl functionalized imidazolium ionic liquid crystals have been used as SSCMs [297]. Again, this study demonstrated the performance advantages of applying a liquid crystalline phase in ss-DSSCs. Here, the LCs performed both carrier mediation for dye regeneration and hole transportation in the LC phase. An innovative approach was applied to obtain three-dimensional (3D) self-assembled structures, which involved engineering imidazolium-based ionic LCs, which could retain a liquid crystalline phase at outdoor temperatures [297]. The SSCMs in this study comprised two variants of double-alkyl functionalized ionic liquids, one being 1,3-didecylimidazolium triiodide (1) and the other 1,3-didodecylimidazolium triiodide (2). Both of the ionic LCs were self-formulated and subsequently investigated in the cells. Devices that employed an electrolyte that is only composed of the single-component ionic liquid exhibited solar-to-electrical conversion efficiencies of 1.5% under full sunlight illumination for outdoor conditions, and 3.9% under quarter sun illumination for similar conditions [297]. These were considered to be good values based on past results and since no optimizations were performed, such as implementing additives. At the time of reporting, state of the art ss-DSSCs yielded PCEs <1% at room temperature using single-component solid state conductors in isotropic liquid phase [297]. 

The same study demonstrated that efficient dye regeneration by hole-transportation was only possible in the smectic-C phase combination of the two LCs mentioned [297]. 1,3-didodecylimidazolium triiodide had better diffusivity and ionic conductivity than 1,3-didecylimidazolium triiodide [297]. Thus, an ss-DSSC with the former LC was compared to a state-of-the-art liquid ACN-based DSSC, which had 6.3% efficiency in hysteresis testing, from 25–120 °C at a 2.5 °C min.^−1^ heating and cooling rate under full sun outdoor conditions. The state-of-the-art cell did not survive the test due to its volatile electrolyte, resulting in 2.4% efficiency post-test. However, the ss-DSSC with 1,3-didodecylimidazolium triiodide withheld its initial properties of 0.7–1.5% energy conversion efficiency, along with a *V_OC_* ranging from 0.5–0.46 V and *J_SC_* ranging from 6.3–8.3 mA cm^−2^ [297].

In 2014, nanostructured liquid-crystalline ion transporters were employed as electrolytes for ss-DSSCs [298]. New solid-state conductors comprising two-component LCs with a carbonate-based mesogen (1) and a two-dimensional self-assembling ionic LC (2), which was suitable for I^–^/I_3_^–^ based redox mediation, were introduced [298]. The SSCMs proved to be non-volatile and they exhibited liquid crystalline phases over a broad temperature range. Three different combinations of (1) and (2) were tested, amongst which, 1/2-I_2_ with 60% of (1) showed the smectic phase and was thus selected for hole propagation. DSSCs containing these SSCMs in conjunction with a TiO_2_/D35 surface exhibited remarkable photovoltaic performance, with *V_OC_*s of 0.78 and 0.58 V, *J_SC_*s of 4.3 and 8.2 mA cm^−2^, *FF*s of 36 and 52, and PCEs of 1.3% and 2.2% at 30 °C and 90 °C, respectively. The DSSCs were operational at up to 120 °C. These results clearly indicated that the innate characteristics of LC-based SSCMs allow for better interfacial exchange of ions at the PE/electrolyte interface, which is believed to suppress the recombination electrons. This work emphasized the extraordinary features of ionic liquid crystal based ss-DSSCs [298].

A later study on nanostructured LC electrolytes developed efficient and stable quasi-solid-state DSSCs [299]. The best performing electrolyte in this work operated in an isotropic region rather than the smectic phase, unlike in previous studies. Therefore, the electrolyte was more or less a quasi-solid rather than in a solid phase [299]. Two different types of ionic liquid crystal assemblies for iodine doped 1-ethyl-3-methylimidazolium iodide (EMII) electrolytes were synthesized. The first type consisted of iodine doped imidazolium ionic liquids with carbonate-terminated mesogenic compounds non-noncovalently bound as a two-component mixture (assembly type i). The second type consisted of a covalent imidazolium moiety that was doped with iodine and assembled as a single-component mesogenic compound (type ii). The mesogenic compounds in the LC-based SSCM type ii were synthesized with flexible oligooxyethylene spacers that formed the bond between the mesogenic and the polar species. This covalent interaction within the SSCM inhibited crystallization, which lead to enhanced thermal stability for ion transport [299]. The type i SSCM was designed for efficient ion transport, whereas the type ii LC-based SSCM was designed to improve the thermal stability in the liquid crystal phases. As a result, a ten-fold higher diffusion coefficient for the I_3_^–^ ions was observed for the noncovalent type i relative to the covalent type ii SSCM [299]. 

DSSCs with type i LCs with EMII electrolytes demonstrated PCEs of up to 5.8% at 30 °C and 0.9% 120 °C. In contrast, DSSCs with the covalent type ii electrolytes exhibited a remarkable increase in conversion efficiency of up to 2.4% at 120 °C, which was 2.5 times higher than the noncovalent assembly. The type ii SSCM possessed oligooxyethylene spacers in the compound, which extended the liquid crystalline phase temperature range, as well as enhanced the mass transport properties of the electrolytes at higher temperatures. Especially, at temperatures above 90 °C, the devices with the type ii electrolyte had far superior performance to those with the noncovalent type i electrolyte. Moreover, all of the ionic liquid crystal-based DSSCs demonstrated outstanding long-term stability, retaining a PV performance between 90–100% for over 1000 h. Such novel electrolyte compositions enable the development of DSSCs with the capability to efficiently convert light to electricity in a wide range of temperature conditions [299]. 

Very recently, Wang et al. introduced an innovative ionic liquid crystal as an SSCM, which contained hexylimidazolium (HII) that was prepared via in-situ polymerization at 40 °C [300]. The HII ionic polymer had structural properties similar to those of alkylimidazolium iodide, with cations in the polymer main chain meaning that the polymer could act as a redox mediator. The incorporation of 1,3-dimethylimidazolium iodide (DMII) in the HII ionic polymer resulted in a DMII/HII ionic polymer, which greatly improved the conductivity of the electrolyte. These were combined according to the weight ratio (0.7:1) [300]. SS-DSSCs that were based on this SSCM achieved an impressive PCE of 6.55% under full sun irradiance. They also demonstrated high stability, retaining 89% of the initial performance for 30 consecutive days at room temperature [300].

Subsequently, nanofibers of poly(3,4-ethylenedioxythiophene) (PEDOT NFs) were used as a catalytic material for CEs in conjunction with dimethyl imidazolium iodide (DMII). This improved both the electrical conductivity and catalytic activity [301]. The improvement was not only attributable to the PEDOT NFs at the CE, but also to re-doping with DMII as the redox mediator. This combination produced efficient ion exchange due to the enhanced doping concentration of I^–^ ions. The re-doping of PEDOT NFs with DMII increased the mobility of the SSCM more than 18 times when compared to undoped PEDOT NFs with the pre-doped dodecyl sulphate anions (DS^–^) already present due to the polymerization process [301]. This improved conductivity was a result of higher linearization, mitigated aggregation, and refined crystallinity of the PEDOT chains. The catalytic activity also improved because of better compatibility and a greater effective surface area as a result of substituting the sticky DS^–^ ions with the more rudimentary and smaller I^–^ ions from DMII on the surface of the PEDOT NFs. Consequently, the charge-transfer resistance along the edge between the electrolyte and the PEDOT NFs CE was significantly reduced. DSSCs that were based on these achieved an ECE of 8.52%, outperforming the devices with Pt CEs, which had an 8.25% energy conversion efficiency under full sun conditions [301].

Several studies have investigated the influence of replacements and modifications to the imidazolium ring. The ester-functionalized imidazolium conductors have shown good SSCM electrolyte conductivity (5.76 × 10^−3^ S cm^−1^) [302]. DSSCs incorporating these had a reasonably high efficiency of 6.63% and excellent long-term stability, since this value did decrease during 1000 h of continuous light soaking [302].

Similarly, modifying the imidazolium ring by the addition of a propargyl group enhanced the conductivity 40,000 times in comparison to a pre-existing alkyl-substituted imidazolium ring with iodide [303]. A solid-state electrolyte with such intricate modifications was able to achieve a PCE of 6.3%, and it had respectable long-term stability under illumination at AM 1.5 for 1500 h.

Interestingly, a substituted ester group as compared to the methyl bound imidazolium ring resulted in a superior PCE of 7.45%, and a staggering increase in the ionic conductivity of 12–116 µS cm^−1^ [290].

Yet another substantial study resulted in even better performance via doping succinonitrile (SN) molecular plastic crystals in trialkyl-substituted imidazolium iodide salts. This high performing SSCM with superior ionic conductivity (2–4 mS cm^−1^) resulted in a ss-DSSC with a PCE of 7.8% under full sun AM 1.5G illumination [304].

The performance of ss-DSSCs that are based on SSCMs is comparable to certain state-of-the-art DSSCs applying ionic liquids as solid-state alternatives to other electrolytes. These solid-state ionic materials have significantly improved in recent years and the PV performance of devices employing these is rapidly approaching that of the liquid electrolyte DSSCs. SSCMs have not received the same attention as hole-transporting materials, but certainly qualify as promising substitutes for traditional solvent-based electrolytes due to their exceptional features, as highlighted in this section. SSCMs could be a worthy choice for fabricating DSSCs that can function efficiently and stably at high temperatures. Further advances in these materials will likely promote the realization of SSCM-based devices as a separate category of DSSCs that are capable of operating efficiently under extreme conditions.

#### 4.3.2. Inorganic HTMs

In conventional DSSCs, the I^–^/I_3_^–^ redox couple between the mesoporous TiO_2_ layer and the CE is usually considered to be an electron-transporting layer. In ss-DSSCs, the major difference is that the solvent-based redox electrolyte is replaced with a p-type semiconductor material, which is referred to as a hole-transporting material (HTM) [96,97,98,320,321,322,323].

In general, HTMs should not be considered electrolytes, but rather semiconductors. In conventional DSSCs with electrolytes, charge carrier transportation between the PE and CE occurs via ionic transport. In contrast, charge carrier transportation in HTMs takes place via electrons or holes (hole hopping amongst the adjacent molecules or components as in semiconductors), therefore, by definition, it is an electronic transport [323,324]. 

Although HTMs mainly rely upon electron/hole transport, they can be doped with some salts to also introduce slight ionic conductivity, which can substantially compensate for local charge insufficiencies [14]. HTMs that are incorporated within ss-DSSCs must satisfy certain key requirements, including:

(a) Efficient reduction of the sensitizing dye (after it has injected electrons in the TiO_2_) by transferring holes to it. For optimal reduction, it is imperative that the upper edge of the valence band of the HTMs should be present right above the ground state of the sensitizer [36,97,320,323].
(b) Demonstration of efficient pore-filling in the mesoporous medium (for example, TiO_2_) [36]. The inability to fill the pores of the mesoporous TiO_2_ layer is one of the foremost reasons for deteriorating device performance [36,320,325,326].
(c) High hole mobility is desired, as lower hole mobility is believed to be another limiting factor for the device performance of ss-DSSCs [36,320,327,328].
(d) The HTM should also be adequately transparent in the visible light range; therefore, it must have a wide band gap and it should not negatively affect the sensitized dye, either by dissolving it or by causing degradation during the depositing process [36,320,322,323].

Based on the above criteria, only a handful of inorganic p-type materials are suitable as HTMs in ss-DSSCs [36]. Industrially well-known wideband HTMs, such as silicon carbide and gallium nitride, cannot be utilized, since the high temperature processing related to their deposition degrades the sensitized dyes that are anchored on the TiO_2_ electrodes [320]. As alternatives, copper-based p-type materials, such as CuI, CuBr, and CuSCN, have been investigated [305,329,330,331]. These can either be spin casted from solutions or deposited through vacuum processes, and they still retain sufficient mobilities for hole conductivity [322].

The first ever report of an HTM and a corresponding ss-DSSC was by Tennakone et al. in 1995 [305]. The study introduced CuI as the first p-type material to serve as an HTM. It was successfully integrated into an ss-DSSC comprising a mesoporous TiO_2_ layer with a monolayer of cyanidin pigment [305,323]. CuI was shown to be a good candidate material, because it had a wide band gap (3.1 eV), along with excellent solubility in ACN prior to deposition [323]. SS-DSSCs with this configuration yielded a PCE of no more than 0.8% under 800 W m^−2^ intensity illumination in outdoor conditions, with a *V_OC_* of 375 mV and *J_SC_* of 2.5 mA [305]. Weak adhesion and insufficient pore filling in the TiO_2_ layer by the CuI as a result of its fast crystallization is mainly attributed to their weak power efficiency. Additionally, cells that were fabricated with CuI were observed to be very susceptible to moisture [305,332].

In a follow up study, taking advantage of the valence band edge of CuI (–5.3 V vs. the vacuum level), which closely matches the HOMO level of the ruthenium (Ru) bipyridyl dye, the previously used cyanidin pigment was replaced with a ruthenium dye, which greatly improved the solar-to-electrical conversion efficiency (up to 4.5% under one sun illumination) [306,332,333]. The same co-workers also reported other small improvements that arose, for example, from the addition of minute amounts of 1-methyl-3-ethylimidazolium thiocyanate (MEISCN) or triethylamine hydrothiocyanate (THT) to the coating solution for inhibiting the crystallized growth of CuI [332,334,335]. This improved the interactive exchange between CuI and the sensitized TiO_2_ layer on the photoanode [334,335].

Mixing ZnO nanoparticles into TiO_2_ nanoparticles has also been found to promote the electrical contact and suppress the recombination losses, resulting in an efficiency <4% [332]. More interestingly, still, introducing a blocking layer of MgO before sensitizing the PE, to effectively block the photogenerated holes to the CuI layer, has been found to increase the solar-to-electrical conversion efficiency by up to 4.7% [336]. In this setup, the imperfect contact between the dye and the HTM was considered as the main limitation for further efficiency enhancement [336,337]. This hurdle was overcome by utilizing a cis-dithiocyanate-bis (2,2′-bipyridyl-4,4′-dicarboxylate) ruthenium (II) sensitizer, which showed superior contact of its NCS ligands that were bound to the CuI surface. The use of this sensitizer led to increased conversion efficiency to 6% (under 5 mW cm^−2^) [306].

Furthermore, the use of a CuI as a hole conductor utilizing the NCS ligand of GuSCN (guanidine thiocyanate) in combination with PEDOT:PSS CEs in a ss-DSSC was also demonstrated [338]. The study exhibited dramatic improvements in the conversion efficiency to 7.4% under full sunlight illumination, which is, to date, the highest efficiency known for a CuI HTM-based DSSC [338,339].

However, efficiencies that were reported in subsequent studies did not improve any further, and they ranged from 1–5.5% at most [340,341,342,343,344]. In 2014, Amalina and Rusop conducted an investigation into the properties of CuI thin films [340]. They employed a novel-mist atomization method to deposit a CuI film over the sensitized photoanodes, which indicated the potential suitability of CuI thin films as p-type hole conductors for ss-DSSCs [340]. The best device efficiency was reported to be 1.05% under full sunlight illumination, which was achieved by optimizing both TiO_2_ porosity and the concentration of the CuI solution to 0.05 M. The investigation concluded that the matching of the nano sized CuI particles with the size of the porous structures of TiO_2_ layer was the key factor that contributed to better device efficiency [340].

A later study demonstrated the doping effect of I_2_ for CuI HTMs, and investigated the device performance of ss-DSSCs while using different amounts of iodine doping, by varying the I_2_:CuI weight ratio [341]. High electrical resistivity resulted from increased iodine doping as compared to pristine thin films of CuI. This was a consequence of surface traps that were created by iodine doping. The cells with un-doped CuI as the HTM demonstrated the highest efficiency, of 1.05% under one sun illumination. This reflected the corresponding resistivity values of CuI thin films. The cell containing the highest amount of doping with 40 mg I_2_:CuI demonstrated the lowest PCE of 0.45%. The study further concluded that the size of the CuI crystals and the degree of crystallization of the CuI-based HTMs critically affects the solar cell performance [341].

Taha et al. demonstrated the effect of pulse laser deposition (PLD) for both TiO_2_ and the CuI thin films on the performance of ss-DSSCs [342]. The thin films were fabricated by PLD under 3 × 10^−3^ mbar of vacuum pressure and annealed at 450 °C. The laser used was a 1064 nm wavelength with varying (200, 500, 800 nm) pulses for deposition. The study found a direct relationship between the photocurrent from the TiO_2_ and the laser pulses that were used during fabrication; the more laser pulses applied, the higher the photocurrent at the TiO_2_ layer. This led to a leap in the conversion efficiencies of TiO_2_, from 2.115% up to 5.654% and for CuI from 1.73% to 5.19% upon increasing the number of pulses from 200 to 800 [342].

In 2016, Konno demonstrated the difference between fabricating the DSSC electrodes by pulse electrodeposition and continuous electrodeposition [343]. The cells that were prepared by pulsed electrodeposition under illumination had a collection efficiency of 3.28%, whereas the cells that were prepared by continuous electrodeposition had a PCE of only 0.75%, both in the absence of THT growth inhibiter and under simulated AM 1.5G illumination. The same study also demonstrated that CuI films that were grown by the combination of pulse electrodeposition (without THT), followed by solution casting, demonstrated an even higher efficiency of 3.85% under similar illumination conditions [343]. This study was especially interesting, since earlier work has shown the characteristic efficiency of ss-DSSCs to drastically improve by using THT (PCE increases from <1 up to 4%) [305,306,332,333].

Hanif et al. in 2017 modified CuI by adding TMED (tetramethylethylenediamine) and NH_4_SCN (ammonium thiocyanate) in the fabrication of ss-DSSCs [344]. The study included several volume variations of TMED (0.1, 0.2, and 0.4 mL) and mixing ratios of TMED:NH_4_SCN (1:1, 1:2, 2:1)_._ It was observed that maximum improvements resulted from using both of the additives together, according to a 2:1 ration of TMED:NH_4_SCN. This resulted in improved optical properties through an increase in the band gap energy from 2.38 to 3.79 eV. Similarly, there were improvements in conductivity. CuI with simple TMED (0.4 mL) exhibited a conductivity of 0.29 S m^−1^, whereas the corresponding value with added TMED:NH_4_SCN (2:1) was 0.39 S m^−1^ and that with pristine CuI was 0.26 S m^−1^. The fabricated ss-DSSC consisted of nanorods of TiO_2_ on the photoanodes that were sensitized by N3 dye, followed by the HTM layer, and lastly platinum as a CE [344]. The performance of ss-DSSCs with the HTM based on pure CuI resulted in a PCE of 0.46%, whereas the cells containing CuI-TMED had an efficiency of 0.9%, and those with CuI-TMED:NH_4_SCN exhibited the highest efficiency of 1.52%. Hence, the highest efficiency was 3.28 times higher when CuI was dissolved in TMED:NH_4_SCN than when pure. Therefore, the combination of TMED and NH_4_SCN with CuI is integral to improved optical and electrical properties of ss-DSSCs employing CuI [344].

Copper thiocyanate (CuSCN) is an alternative inorganic HTM that may have the potential to replace CuI due to its better stability and unique chemical robustness that arises from its polymeric structure [36,329,345,346,347]. Nevertheless, poor hole-conductivity and a poor reduction rate for the already oxidized dye molecules mean that efficiencies with this material have generally been lower [329,345,348,349]. Amongst the early studies, CuSCN dissolved in n-propyl sulfide ([C_3_H_7_]_2_S) that was deposited on an Ru-dye coated film gave efficiencies of 1.25–2% for fabricated ss-DSSCs [329,350]. Thus, CuSCN alone was insufficient as an HTM. Therefore, a doped version of the HTM with SCN was investigated, which showed decreased band gap energy, which typically ranges from acceptor levels of 3.6 eV (CuSCN) to 1 eV (SCN doping) [348]. Doping with SCN enhanced the efficiency from 0.75% to 2.39%. The intrinsic low efficiency of CuSCN-based devices was probably due to its inadequate fill factors and to deficits in photocurrent densities [351]. This was attributed to faster recombination reaction rates in this HTM than in the liquid electrolytes. Further improvements in the efficiency of CuSCN as an HTM would require the reduction of these recombination rates. This could be achieved by either the addition of surface passivation layers or by significant escalation of electron transportation rates within the mesoporous titania layer on the photelectrodes [349,351]. In recognition of this, a study was conducted to improve the hole mobility by including Cu (II) sites coordinated with triethylamine within the CuSCN HTM structure. Hole mobility was enhanced 1.42 × 10^4^ fold, which improved the native mobility of 0.01 S m^−1^ of the original CuSCN to an extremely enhanced mobility of 1.42 S m^−1^ for the modified CuSCN [307]. Devices employing the modified CuSCN delivered a PCE of 3.4% under full sunlight illumination [36,307].

In another study, a moderately efficient ss-DSSC with a PCE of 5.1% for an active surface area of 28 mm^2^ was achieved via the electrodeposition of CuSCN thin films and nanowires [308]. The performance of these ss-DSSCs was compared to that of the traditional CuSCN-based ss-DSSCs. The results demonstrated that electrodeposition of the HTM caused nano-structuring, which led to efficient hole transport. The study also investigated the impact of the HTM thickness on device performance. It was established that the CuSCN nanowire array thickness must not exceed the active layer thickness of the sensitized PE, in order to achieve high performance [308]. High performance resulted in the form of remarkable *V_OC_* (more than 900 mV), along with an impressive fill factor of up to 60%. The possibility of applying the CuSCN HTM thin films and NWs directly with this method, without the need of an annealing process, could result in cost reductions in the fabrication of future DSSC devices [308].

Amongst the other Cu-based HTMs, a recent entrant, i.e., bis(2,9-dimethy-1,10-phenanthroline) copper ([Cu(dmp)_2_] was accidentally discovered [186]. Several other copper redox mediator models were already known for their fast electron-transfer mediation characteristics in electrolytes, with the examples including bis (1,10-phenanthroline) copper ([Cu(phen)_2_]), [(−)-sparteine-N,N′](maleonitriledithiolato-S,S′), copper ([Cu(SP)(mmt)]), and bis(2,9-dimethy-1,10-phenanthroline) copper ([Cu(dmp)_2_]) [100,352]. Sterically constrained [Cu(dmp)_2_] was found to have the fastest electron mediation rate (23 M^−1^ s^−1^) among these, giving rise to an energy conversion efficiency of 2.2% under a low light intensity (200 W m^−2^). This was the highest reported conversion efficiency at that time [352]. Exploiting this rapid electron self-exchange, the [Cu(dmp)_2_] molecules were tested for their potential as a HTM in ss-DSSCs [186]. The ss-DSSCs were fabricated by the evaporation of the high vapour pressure solvents from the liquid [Cu(dmp)_2_] electrolyte in ambient air. Extraordinarily, these had a *J_SC_* of 13.8 mA cm^−2^, even surpassing that of liquid electrolyte-based DSSCs (9.4 mA cm^−2^) [100,186]. The PCE of the ss-DSSCs was 8.2% under full sun irradiance, which surpassed all previous counterparts made using CuI and CuSCN. The ss-DSSCs incorporating this new HTM were termed ‘zombie cells’ [186].

Further refinements in 2017 resulted in a record breaking hole-transporter that was composed of a mixture of Cu[(4,4′,6,6′-tetramethyl-2,2′-bipyridine)_2_](bis(trifluoromethylsulfonyl)imide)_2_] [Cu(tmby)_2_](TFSI)_2_] and Cu(4,4′,6,6′-tetramethyl-2,2′-bipyridine)_2_(bis(trifluoromethylsulfonyl)imide)] [Cu(tmby)_2_](TFSI)], incorporated in an amorphous state. This mixture conducted holes by rapid hopping upon infiltrating a 6.5 μm thin mesoscopic TiO_2_ layer, achieving high efficiency [100]. The time constants for electron injection and regeneration from the Y123 sensitizer by Cu(I) were 25 ps and 3.2 μs, respectively, hence the dark reaction was 1.28 × 10^6^ orders of magnitude slower. This resulted in a PCE of 11% for this stable ss-DSSC under full sunlight illumination [100].

Cesium tin iodide (CsSnI_3_) is an alternative to copper-based HTMs, especially with certain modifications. B-γ-CsSnI_3_, in particular, has already gained significant interest due to the potential for its use in ss-DSSCs [31,353,354], Schottky solar cells [355] and other optoelectronic devices [356,357,358]. These prospective applications are supported by the exceptional properties of CsSnI_3_, which include high a p-type metal-like conductivity of around 200 S cm^−2^ [31,353,354,355,356,357,358,359,360,361], hole mobility exceeding 585 cm^2^ V^−1^ s^−1^, a direct band gap of approximately 1.3 eV, and high near-infrared luminescent emissions of around 950 nm [36].

In 2012, Chung et al. employed CsSnI_3_ for fabricating ss-DSSCs [31]. The CsSnI_3_ solution used was able to penetrate into the compact titania nanopores at the molecular level, ensuring strong interfacial contact with sensitizer molecules as well as the TiO_2_ layer. The HTM exhibited an optimum band gap of 1.3 eV, improving light absorbance in the visible range as well as at the lower wavelength edge of the light spectrum. This far exceeded the functional performance of conventional DSSCs in a similar spectral region. For devices that were fabricated with pure and undoped CsSnI_3_, the corresponding ss-DSSCs had PCEs of only 3.72% under standard full sun irradiance conditions. However, doping CsSnI_3_ with 5% SnF_2_ increased the conversion up to 6.81%. In addition, fluorine plasma pre-treatment of the TiO_2_ layer on the PE and applying photonic crystals on the CE, further improved the device performance, yielding an even higher energy conversion efficiency of 8.51% under standard full sunlight illumination [31].

In 2014, Lee et al. applied Cs_2_SnI_6_ (a dimer of CsSnI_3_) in ss-DSSCs [292]. The 4^+^ oxidation state of the Sn in Cs_2_SnI_6_ makes this HTM less vulnerable to both natural moisture and air than its predecessors, such as CsSnI_3_ and CH_3_NH_3_SnI_3_, which both possess highly reactive Sn (tin) in the 2^+^ oxidation state. Treatment in an inert and chemically inactive environment is required for the fabrication of DSSCs with these reactive materials. Devices utilizing Cs_2_SnI_6_ as the HTM in combination with a Z907 sensitizer, and fabricated in air under ambient moisture conditions, demonstrated a PCE of 4.7% at 100 mW cm^−2^ irradiance. SS-DSSCs with other mixes of dye sensitizer, such as N719, YD2-o-C8, and RLC5, achieved even higher efficiencies, nearing 8% [292].

The same year, Ma et al. demonstrated similar ss-DSSCs while employing a variant of the inorganic HTM CsSnI_2.95_F_0.05_, which was specifically selected to improve the interconnection between the HTM precursor and the photoanode TiO_2_ nanorod array [309]. They also quantified the correlation between the HTM precursor that was adsorbed onto the photoanode based TiO_2_ array and hole injection from the dye to the HTM in ss-DSSCs. A PCE of 5% was achieved under full sunlight illumination. Under 14.2 mW cm^−2^, the average conversion efficiency was 7.7% for a batch of fabricated cells and among these the best performing device had a PCE of 9.8%. The study also developed a physics-based device-level model for this type of ss-DSSC. This model described the diverse kinetics that occur between the active TiO_2_ layer, the dye, and the HTM, instead of employing empirical kinetic equations alone [309].

In 2016, Kaltzoglou et al. demonstrated the use of “defect” perovskites Cs_2_SnX_6_ (X = Cl, Br, I) in DSSCs as HTMs, and described their vibrational, optical, and other properties [362]. The Cs_2_SnX_6_ perovskites were first synthesized and characterized, used in the fabrication of perovskite cells, and then introduced as HTMs in DSSCs [362]. The ss-DSSCS were composed of mesoporous TiO_2_ PEs sensitized with either organic or metal–organic dyes. DSSCs with Cs_2_SnI_6_ as the HTM in combination with Z907 dye displayed a peak performance of 4.23% PCE under full sun irradiation. Electrochemical impedance spectroscopy indicated that this PCE resulted from both efficient hole extraction at the perovskite-Pt interfaces and proficient charge transport across the Cs_2_SnI_6_ HTM.

Recently, Lee et al. reported the use of the air-stable semiconducting iodosalts, Cs_2_SnI_6−x_Br_x_, as stable and environmentally safe potential HTMs for solar cells [363]. A range of values of x higher than 3 for compounds of Cs_2_SnI_6−x_Br_x_ provided the desired bandgap range of 1.3 eV to 2.9 eV, which is suitable for DSSC design. As explained above, the 4^+^ oxidation state of Sn within these compounds provides stability and resistance to moisture degradation during device fabrication and operation. This study considered in detail challenges for both synthesis and solution processing for the application of this HTM. A two-step solution synthesis method was established, wherein a well-defined CsI crystalline film was composed as the first step. The second step represented a chemical reaction with a solution of SnI. A series of Cs_2_SnI_6−x_Br_x_ films was produced as a result of adjustments that were made at each processing step and based on detailed structural, electrical, and optical characterization. The study emphasized the importance for optimal performance of achieving a stoichiometrically accurate compound during cell fabrication. Cells employing the compound with x = 2 as an HTM demonstrated an energy conversion efficiency of 2.1% under 100 mW cm^−2^ AM 1.5G simulated solar illumination [363].

Very recently, Kapil et al. conducted a study to investigate the intrinsic properties of Cs_2_SnI_6_ and to fabricate stable and environmentally safer solar cells [364]. Cs_2_SnI_6_ was already an established p-type semiconductor with a reasonable stability, and it had already been utilized successfully as an efficient HTM. However, the nature of the majority carrier for this HTM was still unclear. Therefore, the study explored the fundamental material characteristics of this HTM in considerable detail, and its potential as both a light harvester and a facile electron HTM. Its high absorption coefficient of 5 × 10^4^ cm^−1^ at 700 nm wavelength translated into a 0.2 μm penetration depth of light at the same wavelength. This value is similar to that of the conventional Pb based solar cells.

The deposition of Cs_2_SnI_6_ via spray-coating in combination with spin-coating was presented as a solution to the challenges of preparing impurity free and dense thin films of Cs_2_SnI_6_ by solution processing [364]. Transmission electron microscopy (TEM) investigations revealed the presence of two emission peaks at 710 and 885 nm in the prepared Cs_2_SnI_6_ thin films, which clearly indicated the coexistence of quantum dot and bulk parts in the HTM. Time-resolved photoluminescence (PL) and transient absorption spectroscopy (TAS) demonstrated fast decay kinetics in the picoseconds (ps) to nanoseconds (ns) time range. The mobile charge carrier lifetime was over 300 ns and the charge decay was slow (up to 20 μs). The mobile charge carrier lifetime was measured by time-resolved microwave photoconductivity decay (MPCD) and the charge decay was measured by nano-second transient absorption spectroscopy (ns-TAS).

Hence, inorganic HTMs, and especially CsSnI_3_ and CuSCN, have shown remarkable recent improvements and achieved high overall efficiencies. However, their long-term stabilities have been infrequently reported and they are either partially or completely missing in the literature. Many of the reported studies have been fixated with improving efficiency, whilst ignoring the aspect of long-term stability, which is a cornerstone of their successful application in ss-DSSCs. Hence, the stability aspects require further investigation for their successful commercial use. Another critical concern to be addressed is the reproducibility of their manufacture. Hence, although the newer inorganic copper HTMs, in particular, are interesting, detailed studies with more analytical data are still required to allow for reliable future applications. 

#### 4.3.3. Organic HTMs

P-type semiconductors with organic components or organic materials behaving like p-type semiconductors can be referred to as organic HTMs. They offer numerous desirable attributes, including facile preparation, relatively low costs, and abundant availability [36,365]. Moreover, most organic HTMs are either polymeric or are soluble or dispersible organic solvents, and they can thus be deposited via cost effective and convenient methods, such as spin coating [294], in situ electrochemical polymerization [366,367,368,369,370,371,372], and photochemical polymerization [373,374]. These methods allow for the HTMs to penetrate the nanopores of the TiO_2_ mesoporous films during DSSC fabrication [36,321].

Apart from applications in DSSCs, organic HTMs can also be customized by chemical methods for use in organic solar cells [373,374], organic thin film transistors [375,376], and organic light-emitting diodes [377,378]. Organic HTMs can be classified into two main categories that are based on their composition, polymeric HTMs, and molecular HTMs [36,321].

One of the earliest studies pertaining to ss-DSSCs incorporated polypyrrole (PPy) as an organic HTM [310]. PPy is actually considered to be an insulator, but its oxidized derivatives are good electrical conductors. The conductivity of PPy can range from 2 to 100 S cm^−1^ depending on the constituents of its derivatives [204,311,379]. The study aimed to exploit in-situ photoelectrochemical polymerization for the application of PPy over mesoporous TiO_2_ films [310]. Improved association between the HTM and the mesoporous titania layers anchored with N3 dye was expected to result from this. However, the ss-DSSCs that resulted from this process achieved an efficiency of just 0.1% under low light intensity of 22 mW cm^−2^. A subsequent study substituted the N3 dye with a ruthenium based complex dye (cis-Ru(dcb)_2_(pmp)_2_) [380], and the old Pt CE with a new carbon-based CE [381]. These modifications resulted in a moderately improved PCE of 0.62% under 10 mW cm^−2^ light irradiance. Upon further investigation, it was discovered that the high density and the black colour of the HTM caused a filter effect, whereby the visible light was absorbed by the polypyrrole, which led to poor performance of the cell [310,380,381]. The conditions and reagents that were used in the oxidation process in the production of PPy have a large impact on conductivity [311]. Nonetheless, the use of PPy as an HTM was short lived due to its dark colour and filtering effect. PPy is now principally referred to, and used as, an efficient metal-free catalytic CE material, which is an alternative to Pt [204,379,382,383,384,385,386]. Porous PPy films that were coated on an FTO glass substrate demonstrate a high surface area for catalytic activity, leading to a small charge transfer resistance (R_CT_), which makes PPy more suitable as a CE material in DSSCs than as an HTM [204,311,379].

Another organic HTM, polyaniline (PANI), was first reported as a possible HTM in ss-DSSCs by Tan et al. [312]. However, the devices that were reported in their study exhibited extremely low output PV parameters, such as *V_OC_* = 310 mV and *J_SC_* = 21 µA cm^−2^ (110 mW cm^−2^). Further investigations from the same group led to the conclusion that a variant of PANI demonstrating an intermediate conductivity of 3.5 S cm^−1^ resulted in the best device performance [387]. The corresponding device achieved a current density of 0.77 mA cm^−2^ and a PCE of 0.10% by optimizing the film morphology of the PANIs, especially in relation to their cluster size.

Another study from the same group demonstrated that incorporating additives, such as LiI and TBP in DBSA (4-dodecylbenzenesulfonic acid) doped PANI, resulted in minor enhancements in performance and yielded a conversion efficiency of around 1.15% [388]. These improvements were attributed to the blocking of the recombination charge transfer along the boundary between the HTM and TiO_2_ layers, while enhancing the wetting of the TiO_2_ films.

Duan et al. incorporated I^−^/I_3_^−^ with poly(ethylene oxide)/polyaniline (PEO/PANI) as solid-state electrolytes [313]. The study aimed to expand and improve the catalysis of I_3_^−^ reduction at the electrolyte/CE boundary and along the entire electrolyte system by decreasing the charge diffusion path length. The HTM was also responsible for dye restoration, which meant the efficient oxidation of I^−^ species and/or corresponding hole migration to the CE. The cell with I^−^/I_3_^−^ that was incorporated in the hole transport configuration of a PEO/1.0 wt % PANI yielded a power conversion efficiency of 6.1%. In similar testing conditions, the conversion efficiencies of cells with PEO or PANI alone were 0.8% and 0.1%, respectively [313]. Hence, PANI alone does not represent a good HTM solution for ss-DSSCs. 

The same co-workers also demonstrated DSSCs with PANI-integrated TiO_2_ photoanodes, PANI CEs, and iodide-doped PANI HTMs [389]. The idea was that the photoanode with PANI would relay electrons for dye regeneration and that the PANI-based CEs could support active reduction of triiodide into iodide ions. Similarly, the solid-state PANI HTM could also catalyze the triiodide species, which restricts the charge diffusion path length, while reducing the oxidized sensitizer at anode/electrolyte interface. PV performances with this design were further improved by regulating the assembly process and lithium iodide dosages, resulting in an energy conversion efficiency of 3.1% and good stability under persistent irradiation.

PANI, like PPy, is not regarded as a very efficient organic HTM, but it is usually considered as an alternative CE catalytic material [204,311,365,379]. However, unlike PPy, PANI has been used in conjunction with other polymers as a quasi-solid electrolyte more often than as an HTM [390,391,392,393,394].

Early applications of P3HT (poly 3-hexylthiophene) and P3OT (poly 3-octylthiophene) in ss-DSSCs did not prove fruitful, owing to their somewhat low PCEs (η < 1%). Low PCEs were attributed insufficient pore filling in the mesoporous TiO_2_ layers [314,395,396], which caused low charge separation and even lower collection efficiencies [36,397,398].

The optimization of a number of sequential parameters, including the crystallinity of the TiO_2_, into either an anatase or a brookite structure, its density, and film thickness have been investigated [399]. This led to the use of P3OT as an HTM on a gold CE assembly in combination with an N719 anchored titania layer. This particular device configuration resulted in a PCE of 1.3% and reasonable stability, even after couple of months of storage normal ambient conditions. Further investigation revealed the inclusion of certain additives, such as lithium bis(trifluoromethanesulfonyl)imide (LiTFSI) and TBP in these HTMs, in combination with both D102 [400] and HRS-1 dyes [401], resulted in even better device PCEs of 2.63% and 2.7% under AM 1.5G full sunlight illumination, respectively [36]. Additional enhancement of PCE (to 3.2%) was possible via the penetration of the HTM into vertically aligned titania nanotubes that were sensitized with squarine dye (SQ-1) [36,402].

Song et al. studied the properties of all-solid-state dye sensitized solar cells by employing P3HT as the HTM. P3HT was employed in conjunction with a compact titania layer (c-TiO_2_) as a hole passivation layer, followed by a nanostructured titania film (n-TiO_2_) containing a metal-free organic sensitizer (D149) and a poly(3,4-ethylenedioxythiophene):poly(styrene sulfonate) (PEDOT:PSS) passivation layer [315,403]. The goal was to investigate the effect of stabilization of the DSSC while using a compact, highly ordered, and stable titania system in the active layer in combination with P3HT molecules. In comparison, organic solar cells possessing poly(3-hexylthiophene):phenyl-C61-butyric acid methyl ester (P3HT:PCBM) demonstrate a drop-in photocurrent density *J_SC_* with time, owing to an P3HT domain size. The ss-DSSCs with highly conductive and ordered titania layers that were fabricated by Song et al. achieved a high *Jsc* of 10.0 ± 0.4 mA cm^−2^ and a moderate PCE of 2.7 ± 0.1% under simulated full sun irradiance.

In 2017, Chevrier et al. fabricated ss-DSSCs incorporating an organic dye (D102) with HTMs (P3HT and 2,2′,7,7′-tetrakis-(N,N-di-p-methoxyphenylamine)-9,9′-spirobifluorene (spiro-OMeTAD)) [316]. The study resulted in PCEs of 4.78% for a P3HT-based cell in comparison to 3.99% efficiency for a spiro-OMeTAD-based ss-DSSC under full sun (100 mW cm^−2^) illumination. This demonstrated that P3HT could be a viable, efficient, and low-cost alternative to spiro-OMeTAD for application in solid state PV technology. In this study, P3HT was designed to combine high regioregularity, a medium-range molecular weight, and narrow dispersity. Semi-crystalline domains were formed as a result of annealing the P3HT chains. This led to enhanced hole mobility, photocurrent collection, and a better overall device performance.

Amongst all of the conjugated polymeric (organic) HTMs, poly(3,4-ethylenedioxythiophene) (PEDOT) stands as uniquely distinctive and versatile. Although PEDOT absorbs visible light and thereby decreases the light-harvesting efficiency of dyes, it nonetheless has sufficient transparency in the visible range for PV applications [101,321,322,325] and it possesses high hole conductivity (300–550 S cm^−1^) [404,405,406]. It also possesses outstanding stability in standard conditions, and therefore has been considered as an excellent alternative HTM for ss-DSSCs [36].

Although PEDOT demonstrates high insolubility as a polymer, it still exhibits exceptional conductivity, which outmatches those of polyaniline, polypyrrole, and polythiophene. In 2004, Saito et al. reported an efficiency of 0.53% under full sunlight illumination while using chemically polymerized PEDOT as an HTM [407]. Later on, their configuration was further improved by replacing the dye with a hydrophobic sensitizer and using the electro-polymerization method for HTM deployment, along with numerous doping agents [366,367,369,370,371,372]. A continuation of such efforts led to the formation of ss-DSSC delivering a PCE of 2.85%, which used lithium bis-trifluoromethane sulfonylimide (LiTFSI) as a doping agent [372].

In 2010, Liu et al. designed a thin layered 2,2′-bis(3,4-ethylenedioxythiophene) (bis-EDOT) hole transporting material by utilizing in situ polymerization to generate a modified PEDOT layer over the mesoporous TiO_2_ with the organic dye (D149) as the sensitizer [408]. The corresponding ss-DSSC obtained a good energy conversion efficiency of 6.1% under AM 1.5G illumination, showing a remarkable jump in efficiency when compared to previous studies.

Kim et al. subsequently fabricate ss-DSSCs with a highly conductive PEDOT and transparent (organized mesoporous) OM-TiO_2_ layers, which resulted in an even higher PCE of up to 6.8% under full sunlight illumination [409]. Liu et al. achieved even higher energy conversion efficiencies with a PEDOT HTM that was produced by photoelectrochemical polymerization (PEP), in conjunction with metal-free, indoline-type organic dyes, D149 [408,410] and D205 [411]. PCE reached 7.1% with the latter dye. In this study, PEP was done under a monochromatic light source, contrary to conventional PEP, which is performed under a Xenon lamp. Device performance was observed to be dependent on the wavelength of monochromatic light used during the PEP procedure of the PEDOT preparation [411].

Zhang et al. reported highly efficient ss-DSSCs with PCEs of 7.11% and outstanding *J_SC_*s of 13.4 mA cm^−2^ [412]. In-situ PEP was used to deposit the PEDOT layer, in conjunction with an effective multifunctional organic dye. The LEG4 dye also operated as a passivation layer, which inhibited charge recombination along the boundary between the sensitizer on the PE and the HTM. The study revealed that an organic sensitizer with well-tuned energy levels and a bulky structure, having a donor-π-acceptor configuration, such as LEG4, in combination with in-situ electrochemically doped PEDOT HTM in ss-DSSCs, leads to an effective dye regeneration and improved photo-charge injection, resulting in high PV performance.

Spiro-OMeTAD has the highest known *V_OC_* (>0.9 V) among all of the organic HTMs [413,414]. Currently, it is the best-known HTM for both ss-DSSCs and perovskite solar cells [413]. Spiro-OMeTAD was first reported by Grätzel et al. in 1998, where it was used with dopants, like N(PhBr)_3_SbCl_6_ and Li[(CF_3_SO_2_)_2_N], along with a dye N719 as the sensitizer, within an efficient ss-DSSC [97]. The overall PCE achieved for the device was 0.74% and the corresponding IPCE value was 33% under standard full sun AM 1.5G conditions [97]. Charge recombination was observed all over the interface of the TiO_2_ and the HTM heterojunction, and it was the leading reason for the low PCE. 

In 2001, Kruger et al. investigated the effect of employing additives, such as TBP and LiTFSI, in spiro-OMeTAD [414]. The study revealed an unexpected 900 mV photovoltage, 5.1 mA photocurrent, and remarkable PCE of 2.56% [414]. Soon afterwards, Snaith et al. conducted a comparative study, making use of numerous molecular sensitizers that are suitable for ss-DSSCs in conjunction with spiro-OMeTAD [415]. This resulted in an improvement in the charge recombination, and a prolonged electron lifetime, by the addition of di-block ethylene-oxide:alkane ligand on the sensitizing dye. The light harvesting capability in the active layer was also increased in the resulting device by replacing the dissipative Au electrodes with reflective Ag electrodes, resulting in a PCE of 5.1% [415].

Another study by Krüger et al. focused on improving the light harvesting properties of cells by mixing silver ions in N719 dye solution [416]. As a result, the ss-DSSC attained a PCE of 3.2%. The dye N719 was substituted with many dyes in consequent studies to further enhance the device performance. One of these studies utilized an amphiphilic ruthenium dye Z907 with a hydrophobic spacer that yielded a PCE of 4% [417]. Another study utilized a pure organic metal free indoline dye (D102) that also yielded a PCE of 4% [418]. Yet another related study utilized a ruthenium complex based sensitizer C104 and delivered an improved PCE of 4.6% [419]. Finally, the high molar extinction coefficient D−π–A organic dye C220 yielded a clearly improved PCE of <6% at full sun irradiance [420]. Therefore, it is evident that, whilst spiro-OMeTAD in its original form suffers from low conductivity, with certain tweaks, its performance can be improved [421,422].

In 2011, an exceptional enhancement of the PCE of ss-DSSCs with spiro-OMeTAD was achieved by increasing hole mobility, attaining an efficiency of 7.2% [293]. Here, the ionic mobility of spiro-OMeTAD was improved more than ten-fold by simply including an additive FK102 with a Co^3+^ complex, along with the more efficient D−π–A organic dye Y123.

Another study by Yi et al. produced an encouraging efficiency of 12%, by utilizing a porphyrin dye Y350-based cell with a [Co(bpy)_3_]^2+/3+^-based liquid electrolyte [423]. Corresponding solid state devices that are based on the same Y350 dye in combination with spiro-OMeTAD as the HTM achieved a PCE of 4.8% under synthetic full sun conditions. A sequential optimization was also carried out by co-sensitization of Y350 with Y123, yielding a higher PCE of 6.4% that corresponds to a current density *J_SC_* of 10.8 mA cm^−2^, open circuit voltage *V_OC_* of 887 mV, and a fill factor FF of 0.66. These results were impressive for solid-state devices at that time.

In 2015, Xu et al. employed a low-cost, chlorinated hydrocarbon solvent, called 1,1,2,2-tetrachloroethane (TeCA), as an additive to spiro-OMeTAD [294]. TeCA was shown to readily oxidize spiro-OMeTAD to Spiro-OMeTAD^+^ under ultraviolet light irradiation. Doing so substantially increased the electrical conductivity of the spiro-OMeTAD film. The study systematically investigated the effect of doping spiro-OMeTAD with TeCA on the PV performance of the cells, and revealed that doping not only improves the overall performance of the DSSCs, but also results in better reproducibility when compared to other common p-type dopants. A remarkable energy conversion efficiency of 7.7% was achieved under standard solar irradiation (AM1.5G, 100 mW cm^−2^), which was a record at that time [294]. This successful application of TeCA as an additive for HTMs offered new prospects for the low-cost fabrication of highly efficient ss-DSSCs [294].

In 2016, Zhang et al. published a comparative study of organic sensitizers for DSSCs [424]. Organic sensitizers with the D–A–π–A configuration had superior performance to their D–π–A analogues, such as the Y123 sensitizer [424,425]. This can be attributed to the auxiliary acceptor in the D–A–π–A configuration, which provides better tuning of the molecular energy levels and extends the spectral response towards red wavelengths, thereby improving the photostability [424,425]. The group designed and synthesized three BTZ-based D–A–π–A sensitizers (XY1, XY2, and XY3) by introducing various π-bridges, together with the cyclopentadithiophene (CPDT) unit [424]. The molar extinction coefficient was improved for XY2 by replacing the benzene ring in XY1 with a thiophene unit, resulting in an extension of the absorption response [424,425]. The highest PCE of 7.51% under full sunlight illumination (AM1.5G, 100 mW cm^−2^) was achieved by cells with the XY2 sensitizer and with an extremely thin mesoporous TiO_2_ layer of approximately 1.3 μm and a spiro-OMeTAD HTM [424]. ss-DSSCs that comprised of the XY1 and XY2 sensitizers in conjunction with spiro-OMeTAD displayed average conversion efficiencies of 6.69% and 6.89%, respectively, which outperformed the reference Y123 sensitizer-based cells, whose average efficiency was only 5.77% under similar illumination conditions. A moderate PCE of 5.50% was achieved by the corresponding ss-DSSC even with a small offset existing between the HOMO energy levels of spiro-OMeTAD and the XY3 dye. The lower *V_OC_* of the cell resulted in a loss of efficiency as a result of using this particular sensitizer. 

In 2017, being inspired by their previous works, the same group further formulated and synthesized two more organic D–A–π–A sensitizers employing the CPDT π-bridge in two pyrido [3,4-b] pyrazine featured D–A–π–A dyes, SH3 (CPDT π-bridge) and SH4 (EDOT π-bridge), for ss-DSSCs [426]. A bulky DAP-based indoline donor group was specifically introduced into the dye structure to curtail the carrier recombination that was taking place along the interface of the sensitized TiO_2_ and spiro-OMeTAD. PV characterization revealed that the SH3 sensitized ss-DSSCs achieved a higher PCE of 5.07% with an extremely thin TiO_2_ layer of 0.6 μm under simulated full sun AM 1.5G irradiance, while the ss-DSSC based on SH4 obtained an inferior PCE of 1.69% under the same operation conditions. An intense reduction in molar absorptivity along with overall performance was observed, owing to the presence of a nitrogen atom in the structure of 2,3-diphenylpyrido[3,4-b] pyrazine near the π-bridge, EDOT, in the dye. The EIS measurements were in agreement with the increased charge recombination rates in the cells based on SH4, which resulted in poor charge collection efficiency, low *J_SC_*, *V_OC_*, and thus lower energy conversion efficiency.

A follow-up study further improved ss-DSSC efficiencies with the organic HTM spiro-OMeTAD, by designing and synthesizing two new organic blue coloured dyes, S4 and S5, with indenol [1,2-b]thiophene functionalized triphenylamine as the donor, PP, and quinoxaline as the auxiliary acceptor, respectively [427]. The S5 dye, which contained the quinoxaline unit, displayed a considerably large molar absorptivity of 63,000 M^−1^ cm^−1^ at 600 nm. Moreover, the ss-DSSCs with S5 demonstrated PCEs of 7.81% and 8.25% under the simulated full sun and half sun solar illuminations, respectively, outperforming the conversion efficiencies of previous LEG4-based DSSCs (7.34% under similar operating conditions). These results indicate that high efficiency HTMs, combined with molecular engineering, represent a promising route for the further development ss-DSSCs with blue-coloured dyes. The study also demonstrated the potential for new co-sensitization routes to enhance the optical window, by combining of blue organic dyes with traditional organic dyes of yellow-red colours.

Recently, a similar investigation by Li et al. produced two new quinoxaline-based D–A–π–A type organic dyes, AQ309 and AQ310 [317]. These dyes were designed by employing EDOT and CPDT as π-linker units to the structure of the older D–π–A organic dye LEG4. DSSCs employing the AQ310 sensitizer in conjunction with Spiro-OMeTAD reached an impressive energy conversion efficiency of 8.0% under the standard full sun solar intensity. Like the previous study, these devices also outperformed ss-DSSCs with the LEG4 dye, which had a conversion efficiency of 7.3% under similar conditions. Even better results were produced with the same combination of HTM and organic sensitizer at low light intensity, where a conversion efficiency of 8.6% under half sunlight illumination was achieved. Once again, these results indicated that combining high efficiency HTMs and molecularly engineered dyes is likely to provide further improvements in the efficiencies of ss-DSSCs.

Hence, solid transport materials exhibit high potential efficiency. However, their conversion efficiencies still seem to lag behind those of traditional liquid based DSSCs. Moreover, the long-term stability of these solid-state conductors has rarely been reported, which, once again, raises serious concern regarding the long-term operational performance of the fabricated ss-DSSCs. Table 8 summarizes the best performing ss-DSSCs assembled with solid-state ionic conductors, inorganic and organic HTMs to date.

## 5. Methods for Electrolyte Application from Lab-Sized DSSCs to Large Area Modules

In addition to electrolyte composition, methods for introducing the electrolyte into the cell channel have an immense impact on the overall PV performance of DSSCs [428]. Traditional methods adopted to introduce the electrolyte solution into lab-sized DSSCs are the vacuum back filling method [16,429,430,431,432] and the electrolyte injection filling method [428,433].

In both cases, holes are drilled into the side of the CE, and the PE and CE are then attached to each other with the thermoplastic to construct the cell channel [30,428]. One drop of electrolyte solution is the dispensed on top of the hole, followed by its insertion by creating a vacuum (in the case of a CE with one or two holes) [429,430]. The electrolyte solution can be alternatively introduced by capillarity force when two holes are present in the CE [30,428].

The vacuum back filling and electrolyte injection methods are both scalable and they have been successfully demonstrated in the fabrication of large area DSSC modules [12,434]. In the case of large area DSSCs with two holes, the vacuum is typically applied via one hole and the electrolyte is introduced via the second hole [435,436,437].

These traditional electrolyte-filling methods also have numerous inherent disadvantages despite some advantages. These include possible breakage of glass electrodes during mechanical drilling and additional costs arising from hole-sealing with additional thermoplastic and additional glass cover [10]. Other problems include the uneven distribution of electrolyte solution in the cell and variation in the performance in individual cell channels, due to the molecular filtering effect that is generated by the mesoporous layers in DSSCs [428,438]. These limitations may be potentially reduced by adopting established printing and coating methods for the electrolyte layer, such as screen-printing or inkjet printing [10,209,439].

Screen- or inkjet-printing can contribute to a spatially homogenous electrolyte composition throughout the device. However, to apply these methods, it is necessary to optimize electrolyte viscosity. This also enhances the long-term stability of devices whilst also avoiding electrolyte leakage [10]. Screen-printing and inkjet printing of the electrolyte layer have been successfully developed for both large area [209,439] and lab-sized [10] DSSCs. Both of the methods result in promising features, such as the reduction in overall cell resistances, which appears as a result of additional non-active area in the presence of drilled holes, and it directly contributes to performance loss [10]. Additionally, such methods motivate the investigation of the effect of homogeneous distribution of electrolytes in comparison with traditional electrolyte filling schemes. Other possible methods for producing the electrolyte layer include slot-die coating or blade-coating, which may be adopted for continuous and roll-to-roll fabrication of flexible DSSCs [440].

## 6. Summary and Conclusions

The development of novel electrolyte compositions is a rapidly growing field of research. New entrants, such as copper redox shuttles and other iodine-free electrolytes have shown great potential for producing high performance DSSCs. Copper redox shuttles, for instance, have received worldwide attention due to their high open circuit voltage (*V_OC_*) and very efficient performance under low light intensities. This indicates that highly efficient DSSCs can be fabricated while employing copper redox shuttles for consumer electronics applications, such as electronic appliances and sensors. Nevertheless, electrolytes that were prepared with these novel redox shuttles are normally liquid-based, which results in some issues for the assembly and scaling-up of the DSSCs. Moreover, the long-term device stability of most of these novel redox shuttles-based electrolytes, and especially those that are based on copper, has not been frequently reported and it requires further investigation. However, it is anticipated that the key challenges that are associated with electrolyte deposition and stability and the ionic transport of these novel electrolytes will be addressed in the near future, resulting in the availability of new, affordable DSSCs.

DSSCs are PV devices, which provide a potential pathway to low-cost solar-to-electrical energy conversion and bulk electricity production. Electrolytes are one of the most critical components that determine the success of DSSC commercialization. Their contribution is significant to the charge transfer and dynamics of the DSSCs, thus relaying major impacts on PV performance and on the long-term device stability of solar cells.

As a result of rapid developments in recent years, three categories of electrolytes for DSSCs have emerged: liquid, quasi-solid, and solid-state electrolytes. These are unique to the application in different categories of DSSCs that have evolved with different substrate employment with the passage of time and research. Among several configurations, the conventional glass based DSSCs mostly rely on liquid electrolytes that contain volatile solvents and other organic liquids for high performance. So far, they have displayed the highest solar-to-electrical conversion efficiencies, now approaching 14.3% with innovation and introduction of novel mediator complexes. However, the volatility of the organic solvents employed in the liquid electrolytes cause unavoidable leakages from the DSSC cell assembly, leading to performance degradation over time, and thus limiting the practical applications of DSSCs with liquid electrolytes for prolonged use and device lifetimes.

Nevertheless, this problem can be partly mitigated by employing ionic liquids as solvents. However, ionic liquids cannot provide the similar high PCE performances in return due to mass transport and ion transport limitations as compared to volatile organic solvents, but they can considerably extend the lifetime of the same devices in comparison. Though higher lifetimes or long-term stabilities are achievable by employing ionic liquids, they still are prone to leakages being liquid in nature and still have limited usability for mostly glass based rigid DSSCs. Consequently, quasi solid-state electrolytes were developed for flexible substrates, which can mitigate the leakage problems to a significant level in the case of using flexible polymer or metal substrate applicable in DSSCs. Similar to ionic liquids, they also are not as efficient as organic solvent-based electrolytes. As a result, very high solar-to-electrical energy conversion efficiencies are not yet reported, but they ultimately ensure enhanced stability and longevity for devices. With current trends, quasi solid-state electrolytes are bound to produce a breakthrough result quite soon, as their PCEs are most rapidly improving among all of the other electrolyte categories. Their annual contribution to publications related to DSSCs employing gel based or other quasi solid-state electrolytes has significantly increased, with different mediator functioning quite well, leading to PCEs that are close to 10% achievable already.

Similarly, solid-state ion-conductors have also been shown to best meet the long-term stability requirements. However, their poor electrolyte/electrode interfacial contacts in ss-DSSCs lead to lower conversion efficiencies. By applying HTMs like spiro-OMeTAD, along with organic sensitizers, ss-DSSCs have, however, already yielded an impressive PCE of 8.0%. It is anticipated that further advances in the molecular engineering of organic dyes and their combination with highly conductive HTMs will result in even more stable and higher performance PV devices in the future. The emergence of perovskite solar cells has also opened up the possibility of testing similar HTMs, as developed for those devices in solid-state DSSCs. Therefore, both high-performance and durable solid state DSSCs are highly expected in the near future.

Researchers favor the former over latter, even with such astounding developments undergoing the electrolytes to increase performances in both high efficiency as well as better lifetimes. With more, the number of publications available with higher efficiencies, rather than long term reliability, has inadvertently skewed the research atmosphere. With this review, it has been noted that, as time is passing, more groups are inclined in the race to achieve the next big breakthrough in record efficiencies, rather than focus on the long-term stability, which would perhaps lead to more value to the field of DSSCs in the long run. Publications with high efficiency data almost always skip out on the aging experimentations determining the lifetime worth of the cells. For this reason, such publications make use of volatile organic solvents, rather than stable ionic liquids or gel-based electrolytes.

Consequently, more investigations are needed on novel materials, solvents, and ionic liquids for future electrolytes. Improvements in electrolyte composition remain vital in order to make the large-scale commercialization of DSSCs a viable reality. DSSCs with improved liquid or ionic liquid electrolytes, in combination with stable high-performance dyes and superior catalyst layers, would help to achieve robust stability and longer lifetimes, whilst also maintaining high efficiency. Further investigations are also needed into the interactions between the different components of DSSCs with novel electrolytes of various compositions. An improved understanding of these interactions could yield paradigm changing results.

## Figures and Tables

**Table 1:** Best efficiencies reported for each type of configuration employing numerous electrolytes.

DSSC Configuration	Electrolyte	Dye	Charge Transfer Resistance (Ω-cm^2^)	PCE (%)	ref.
Front illumination					
Glass (conventional)	[Co(phen)_3_]^2+/3+^	ADEKA + LEG4	Not reported	14.3	[15]
Polymer bifacial	I^−^/I_3_^−^	N3	0.66	6.97	[104]
Metal CE–Glass PE	[Co(bpy)_3_]^2+/3+^	Z907	8.0	5.0	[105]
Metal CE–Polymer PE	I^−^/I_3_^−^	N719	2	5.29	[106]
Rear illumination					
Metal PE–Glass CE	I^−^/I_3_^−^	N719	Not reported	9.2	[107]
Metal PE–Polymer CE	I^−^/I_3_^−^	N719	Not reported	8.6	[68]
Special					
Monolithic	I^−^/I_3_^−^	N719	Not reported	9.5	[108]
Solid state	[Cu(tmby)_2_]^2+/1+^	XY-1	Not reported	13.1	[18]

**Table 2:** Highest power conversion efficiencies (PCEs) reported for liquid electrolytes utilizing organic solvents in DSSCs.

Main Solvent of Liquid Electrolyte	Redox Species	Dye	Long Term Stability	PCE (%)	Ref.
Acetonitrile	[Co(phen)_3_]^3+/2+^	ADEKA-1 + LEG4	Not reported	14.3	[15]
Acetonitrile	[Co(bpy)_3_]^3+/2+^	SM315	500 h at 298 k AM 1.5G	13.0	[17]
Acetonitrile	[Co(bpy)_3_]^3+/2+^	YD2-o-C8	Not reported	12.3	[16]
Acetonitrile	[Cu(tmby)_2_]^2+/1+^	D35 + XY1	Not reported	11.3	[19]
Acetonitrile	I^−^/I_3_^−^ (DmPII) GuNCS/TBP	N3	Not reported	11.18	[41]
Acetonitrile	I^−^/I_3_^−^ (DmPII) GuNCS/TBP	C104	Not reported	10.53	[128]
Methoxy acetonitrile	I^−^/I_3_^−^ (DmPII) MAN/TBP	N749	Not reported	10.4	[129]
Acetonitrile + Valerontrile	I^−^/I_3_^−^ (DmPII) GuNCS/TBP	IJ-1	Not reported	10.3	[130]
Acetonitrile + Valerontrile	I^−^/I_3_^−^ (PMII) TBP	Z-910	Unstable	10.2	[109]
Acetonitrile +N-methyl oxazolidinone	I^−^/I_3_^−^	N719	Unstable	10.0	[127]

**Table 3:** Best efficiencies reported with ionic liquid-based electrolytes.

Electrolyte	Dye	Long Term Stability	PCE (%)	Ref.
DMII, I_2_, NBB GuNCS, NaI in BN	C106	1000 h at 60 °C	10.0	[149]
DMII, I_2_, NBB, GuNCS in MPN	C103	1000 h at 60 °C	9.60	[159]
PMII, [MeIm-TEMPO][TFSI], NOBF_4_, LiTFSI, NBB in MPN	D205	800 h at 25 °C	8.20	[160]
PMImI, I_2_, GuSCN, NMBI in MPN	K19	1000 h at 80 °C	8.00	[161]
I_2_, NMBI in PMImI/EMImTCM	Z907Na	672 h at 60°C	7.40	[156]
[Co((MeIm-Bpy)PF_6_)_3_]^2+/3+^, NOBF_4_, GuNCS, TBP in MII/EMINCS	N719	800 h at 30 °C	7.37	[162]
PMII, 4-OH-TEMPO, NOBF_4_, LiTFSI, NBB, in MPN	D205	800 h at 25 °C	7.20	[160]
I_2_, NMBI, GuSCN in PMImI/EMImSCN	K19	-	7.05	[134]
I_2_, 0.5 M NMBI, 0.1 M GuSCN in PMImI/EMImB(CN)_4_	Z907Na	1000 h at 60 °C	7.0	[163]
I_2_, GuSCN, TBP in PMImI/EMImSCN	Z907	1000 h at 55–60 °C	7.0	[158]

**Table 4:** Best efficiencies reported for electrodes based on alternative redox species.

Redox Species	Dye	Long Term Stability	PCE (%)	ref.
[Co(phen)_3_]^3+/2+^	ADEKA-1 + LEG4	Not reported	14.3	[15]
[Co(bpy)_3_]^3+/2+^	SM315	500 h at 298 k AM 1.5G	13.0	[17]
[Co(bpy)_3_]^3+/2+^	YD2-o-C8	Not reported	12.3	[16]
[Cu(tmby)_2_]^1+/2+^	XY1	Not reported	13.1	[18]
[Cu(tmby)_2_]^1+/2+^	D35 + XY1	Not reported	11.3	[19]
[Cu(tmby)_2_]^1+/2+^	Y123	Not reported	11.0	[100]
Ferrocenium/ferrocene	Carbz-PAHTDTT	Not reported	7.5	[191]
Ferrocenium/ferrocene	Carbz-PAHTDTT	Not reported	5.4	[192]
Ferrocenium/ferrocene	Carbz-PAHTDTT	Unstable	4.9	[192]
Ni (III)/(IV) bis(dicarbollides)	N719	Unstable	2.0	[195]
Ni (III)/(IV) bis(dicarbollides)	N719	Not reported	1.7	[194]
Ni (III)/(IV) bis(dicarbollides)	N719	Not reported	1.5	[193]

**Table 5:** Properties of the most common polymer matrices used to prepare gel electrolytes [36,200,222].

Polymer Host	Repeat Unit	Glass Transition Temperature, T_g_ (°C)	Melting Point, T_m_ (°C)
Poly(ethylene oxide) (PEO)	$-{\left({\mathrm{CH}}_{2}{\mathrm{CH}}_{2}O\right)}_{n}-$	−64	65
Poly(propylene oxide) (PPO)	$-\left(\mathrm{CH}\left(-{\mathrm{CH}}_{3}\right){\mathrm{CH}}_{2}O\right)-$	−60	-
Poly(acrylonitrile) (PAN)	$-{\left({\mathrm{CH}}_{2}-\mathrm{CH}\left(-\mathrm{CN}\right)\right)}_{n}-$	125	317
Poly(vinyl pyrrolidone) (PVP)	$-{\left({\mathrm{CH}}_{2}-\mathrm{CH}\left(-{\mathrm{NC}}_{4}H_{6}O\right)\right)}_{n}-$	110	180
Poly(methyl methacrylate) (PMMA)	$-{\left({\mathrm{CH}}_{2}C\left(-{\mathrm{CH}}_{3}\right)\left(-{\mathrm{COOCH}}_{3}\right)\right)}_{n}-$	105	-
Poly(vinylidene fluoride) (PVDF)	$-{\left({\mathrm{CH}}_{2}{\mathrm{CF}}_{2}\right)}_{n}-$	−40	171
Poly(vinylidene fluoride-hexafluoropropylene) (PVDF-HFP)	$-{\left({\mathrm{CH}}_{2}{\mathrm{CF}}_{2}\right)}_{n}{\left({\mathrm{CF}}_{2}\mathrm{CF}\left({\mathrm{CF}}_{3}\right)\right)}_{y}-$	−90	135

**Table 6:** Properties of the most common solvents and plasticizers used to prepare gel electrolytes [14,36,223].

Organic Solvent	Melting Point, (°C)	Boiling Point, (°C)	Dielectric Constant, ε	Viscosity, (cP)
Water	0	100	78.0	0.89
Dimethyl carbonate (DMC)	4.6	91	3.1	0.59
Diethyl carbonate (DEC)	−74.3	126	2.8	0.75
γ-butyrolactone (GBL)	−44.0	204	39.0	1.73
Propylene carbonate (PC)	−49.0	242	65.0	2.5
Ethylene carbonate (EC)	36.4	248	90.0	1.90
Acetonitrile (ACN)	−44.0	82	36.6	0.34
Propionitrile (PPN)	−92.0	97	28.0	0.41
3-methoxy propionitrile (MPN)	−57.0	165	36	1.1

**Table 7:** Best efficiencies reported with gel electrolytes.

Electrolyte Composition	Redox Species	Dye	Long Term Stability	PCE (%)	ref.
Thermoplastic (TPPE)					
PVDF-HFP	I^−^/I_3_^−^	PREDCN2	1000 h at room temperature under 1 sun	10.37	[240]
PVDF-HFP/MPN	TEMPO	MD-153	N/A	10.10	[245]
PVDF-HFP/ACN	[Co(bpy)_3_]^2+/3+^	MK2	700 h under 1 sun	8.70	[244]
PEO/PVDF/MPN	I^−^/I_3_^−^	N719	500 h at 60 °C in the dark	8.32	[205]
PEG/PC	I^−^/I_3_^−^	N719	60 days under ambient conditions	7.22	[212]
PEO/urea	I^−^/I_3_^−^	N719	N/A	6.82	[235]
PEO	I^−^/I_3_^−^	N3	7 days under ambient conditions (unsealed cells)	6.12	[234]
Thermosetting (TSPE)					
MMA-HDDA	I^−^/I_3_^−^	N719	600 h at 60 °C in the dark	10.60	[259]
POE-PAI	I^−^/I_3_^−^	N719	N/A	9.48	[257]
PVA-co-MMA/ACN	I^−^/I_3_^−^	N719	1000 h at 30 °C under 1 sun	9.10	[209]
PVA-co-MMA/MPN	I^−^/I_3_^−^	N719	1000 h at 30 °C under 1 sun	8.61	[209]
PVDF-HFP	I^−^/I_3_^−^	N719	N/A	8.35	[258]
PAMAM-PEO with iodide groups	I^−^/I_3_^−^	N719	N/A	7.72	[247]
BEMA-PEGMA	[Co(bpy)_3_]^2+/3+^	LEG4	1500 h at 60 °C under dark followed by more 300 h at 40 °C under 1 sun	6.40	[252]
Composite polymer electrolytes					
PVDF-HFP/PEO/SiO_2_/EC/PC	I^−^/I_3_^−^	N719	N/A	9.44	[266]
PVA-co-MMA/TiO_2_/ACN	I^−^/I_3_^−^	N719	1000 h at 30 °C under 1 sun	9.40	[209]
PEO/TiO_2_	I^−^/I_3_^−^	N719	300 h at 60 °C under 1 sun	9.20	[206]
PAA/PEG/Graphene	I^−^/I_3_^−^	N719	N/A	9.10	[274]
PVA-co-MMA/TiO_2_/MPN	I^−^/I_3_^−^	N719	1000 h at 30 °C under 1 sun	8.98	[209]
PEO/PVDF/TiO_2_	I^−^/I_3_^−^	N719	500 h at 60 °C in the dark	8.91	[205]
PAN/CNT	I^−^/I_3_^−^	N719	N/A	8.87	[285]
PEO/PVDF/GOS	I^−^/I_3_^−^	N719	500 h at 60 °C under dark	8.78	[208]
PAN/EC/PC/Ac. carbon	I^−^/I_3_^−^	N719	N/A	8.42	[271]
PAA/PEG/PEDOT-graphene/PtCo	I^−^/I_3_^−^	N719	15 days	8.20	[286]
PAN/EC/PC/SiO_2_	I^−^/I_3_^−^	N719	N/A	7.51	[271]
GO/ACN	I^−^/I_3_^−^	N719	N/A	7.50	[273]
POEM/MWCNT/ PVDF-HFP	I^−^/I_3_^−^	N719	N/A	6.86	[272]
SiO_2_	[Co(bpy)_3_]^2+/3+^	D35	N/A	2.60	[267]
Quasi-solid ionic liquid electrolytes					
Phtaloychitosan/PEO/ TPAI/BMII	I^−^/I_3_^−^	N3	N/A	9.61	[282]
Hydrotalcite nanoclay/PMII	I^−^/I_3_^−^	N719	N/A	9.60	[283]
PVDF-HFP/BIm	I^−^/I_3_^−^	N719	1500 h	9.26	[287]
POEI-IS	SeCN^−^/(SeCN)_3_^−^	TA	1000 h	8.18	[288]
ExMMT/PMII	I^−^/I_3_^−^	N3	N/A	7.77	[284]
Al_2_O_3_/MPII	I^−^/I_3_^−^	N719	N/A	7.60	[280]
HPC/MPII	I^−^/I_3_^−^	N719	Outdoor conditions 600 h	7.44	[289]
PEO/PEGDME/ PMImI/TiO_2_	I^−^/I_3_^−^	N719	N/A	7.20	[281]
SiO_2_/MPII	I^−^/I_3_^−^	Z907	N/A	7.00	[278]
PVDF-HFP/MPII	I^−^/I_3_^−^	Z907	N/A	5.30	[279]

**Table 8:** Best efficiencies reported for organic and inorganic hole-transporting materials (HTMs).

HTM	Dye	Long Term Stability	PCE (%)	Ref.
Solid state ionic conductors				
Succinonitrile + DMPII	N719	Not reported	7.80	[304]
MPII + NMBI + LiI + I_2_	MK2	Not reported	7.45	[290]
Succinonitrie + N-methyl-N-butylpyrrolidinium iodide + I_2_	N719	Not reported	6.7	[291]
PMII/I_2_/LiI/EMIm+BF_4_^−^	Metal free organic dye (name undisclosed)	100% original after 1000 h of soaking at 100 mW cm^−2^	6.63	[302]
Inorganic HTMS				
[Cu(tmby)_2_](TFSI_)2_ and [Cu(tmby)_2_](TFSI)	Y123	Stability reported for under 200 h at 500 W m^−2^	11.0	[100]
Fluorine doped CsSnI_3_ + SnF_2_	N719	Not reported	8.5	[31]
Cu(dmp)_2_ HTM	LEG4	Not reported	8.2	[186]
Cs_2_SnI_6_ + Li-TFSI + TBP	a mixture of N719 with YD2-o-C8 and RLC5	Not reported	8.0	[292]
Organic HTMs				
Spiro-OMeTAD + AQ310	AQ310	Not reported	8.0	[317]
Spiro-OMeTAD + S5 dye	S5	Not reported	7.8	[427]
Spiro-OMeTAD + TeCA	LEG4	Not reported	7.7	[294]
Spiro-OMeTAD + FK102 (Co^3+^)	Y123	Not reported	7.2	[293]
PEDOT (PEP)	LEG4	Not reported	7.11	[412]
PEDOT with OM-TiO_2_	N719	Not reported	6.8	[409]
